# Cannabidiol suppresses M2-like macrophage polarization via TRPV4/RhoA/ROCK signaling to alleviate hepatic fibrosis

**DOI:** 10.3389/fimmu.2026.1951332

**Published:** 2026-10-20

**Authors:** Peiling Zhao, Yongmei Liu, Lian Wang, Manqin Yuan, Xiaosu Wang, Ping He, Yuping Wang

**Affiliations:** 1 Clinical Laboratory Center, The Affiliated Hospital of Guizhou Medical University, Guiyang, China; 2 Department of Medical Examination, Guizhou Medical University, Guiyang, China; 3 Department of Clinical Laboratory, Zhejiang Provincial People’s Hospital Bijie Hospital, Guiyang, China

**Keywords:** CBD, hepatic stellate cell, liver fibrosis, macrophage, TRPV4/RhoA/ROCK

## Abstract

**Background:**

Dysregulated buildup of extracellular matrix constitutes a hallmark of hepatic fibrosis, loss of liver architectural integrity, and impaired hepatic functional capacity. Pro-fibrogenic M2-polarized macrophages promote the transdifferentiation of hepatic stellate cells and facilitate fibrotic progression. Cannabidiol (CBD) exhibits immunomodulatory and anti-fibrotic properties. However, whether CBD regulates M2-like macrophage polarization to mitigate hepatic fibrogenesis remains unclear.

**Objective:**

Cannabidiol-driven mitigation of pathological hepatic remodeling was evaluated, with its molecular regulatory mechanisms systematically clarified.

**Methods:**

The modulation of liver fibrosis by cannabidiol was investigated *in vitro* and *in vivo*. A model was created using bile duct ligation (BDL). Anti-fibrotic effects were tested with liver function tests, histopathology, immunohistochemistry, and immunofluorescence. Co-culture systems comprising THP-1-derived macrophages and LX-2 stellate cells were constructed to delineate cannabidiol-mediated regulation of M2-like macrophage polarization and hepatic stellate cell transactivation. Transcriptomic, single-cell transcriptomic, network pharmacology, molecular docking simulation, and dynamic conformational approaches explored cannabidiol’s binding to TRPV4. Western blot, qRT-PCR, flow cytometry, CETSA, Co-IP, and mass spectrometry detected macrophage polarization and under-healing pathway markers. Transwell and colony-formation assays assessed the effects of conditioned medium from CBD-treated M2-like macrophages on LX-2 cell migration, proliferation, and activation.

**Results:**

Cannabidiol mitigated hepatic damage, fibrogenic progression, and inflammatory responses in BDL-operated mice, supported by ameliorated hepatic functional indices and reduced fibrotic biomarkers. CBD inhibited IL-4-induced M2-like polarization in THP-1 cells, resulting in decreased expression of M2-associated markers and pro-fibrotic factors. Mechanistically, CBD may engage TRPV4 and suppress RhoA/ROCK signaling, while concurrently modulating STAT6 phosphorylation. These molecular actions inhibited LX-2 cell activation, as indicated by reduced Col-I and α-SMA expression, along with diminished migration and proliferation. CBD further reduced collagen deposition and M2-associated macrophage infiltration in hepatic tissue. Notably, co-administration of CBD with a TRPV4 inhibitor potentiated anti-fibrotic effects both *in vivo* and *in vitro*.

## 0 Highlights

- Cannabidiol ameliorates hepatic fibrosis.
- It inhibits M2 macrophage polarization.
- The mechanism involves TRPV4/RhoA/ROCK signaling.
- CBD is a potential anti-fibrotic agent via immune modulation.

## 1 Introduction

Persistent liver insult triggers a reparative process known as hepatic fibrosis. Viral hepatitis, alcohol abuse, cholestasis, metabolic disorders, and toxic insults can cause this injury (1). Excessive deposition of extracellular matrix (ECM), formation of fibrotic septa, and progressive disruption of the normal hepatic architecture are the pathological hallmarks of liver fibrosis (2). If not effectively treated, fibrosis may progress to cirrhosis, liver failure, and hepatocellular carcinoma. Several anti-fibrotic candidates are being evaluated in preclinical and clinical studies, but no approved therapy widely reverses established fibrosis. Thus, identifying effective agents and clarifying their molecular mechanisms is an urgent priority.

The profibrogenic activation of hepatic stellate cells (HSCs) serves as the core pathological driver of progressive hepatic fibrogenesis (3). Following sustained hepatic insult, dormant HSCs shift their cell fate and differentiate into actively proliferating myofibroblast equivalents. This cellular transformation is marked by upregulated α-SMA protein abundance as well as boosted production of collagen I and III (4). However, HSC activation is tightly regulated by signals from the hepatic immune microenvironment (5). Among liver immune cells, macrophages—including Kupffer cells and monocyte-derived phagocytes—critically direct pathological fibrogenic remodeling (6). Guided by local microenvironmental triggers, liver-resident phagocytes manifest divergent polarization toward pro-inflammatory M1 or profibrotic M2 phenotypes. M2-like macrophages are generally associated with tissue repair, but sustained M2-like polarization in chronic liver injury becomes maladaptive (7). M2-biased myeloid subsets express CD206 and Arg-1 and release TGF-β and IL-10 (8). These factors promote stellate cell phenotypic switching, increased collagen synthesis, and abnormal ECM accumulation. Clinical evidence further indicates that M2 macrophage infiltration increases with fibrosis severity (9). Thus, targeting M2-associated polarization represents a promising anti-fibrotic strategy.

Cannabidiol constitutes a key non-euphoric phytocannabinoid isolated from Cannabis-originated herbal raw materials. It has anti-inflammatory, antioxidant, immunomodulatory, and anti-fibrotic properties (10). Previous studies show CBD reduces hepatic lipid accumulation and oxidative stress (11). It elicits apoptosis in phenotypically switched stellate cells, restraining fibrogenic tissue remodeling in progressive lung fibrosis by tuning the TGF-β/Smad regulatory network (12). CBD also inhibits M2-like tumor-associated macrophage polarization (13). It alleviates macrophage extracellular trap-mediated liver inflammation and fibrosis. These findings suggest macrophage modulation may help CBD’s protective effects. However, it remains unclear whether CBD directly regulates M2-like macrophage polarization in liver fibrosis or which upstream molecular target is involved.

The transient receptor potential vanilloid 4 (TRPV4) constitutes a mechanically responsive ion channel that mediates Ca²^+^ influx via cation-selective permeability. It responds to matrix stiffness, osmotic pressure, and shear stress (14). An increasing body of evidence shows TRPV4 contributes to fibrotic diseases in multiple organs (15). Genetic deletion or inhibition of TRPV4 reduces fibrotic remodeling (16). In the liver, TRPV4 is linked to hepatocellular carcinoma progression and HSC activation. This occurs partly through AKT-dependent regulation of autophagy and cell survival (17). The role of TRPV4 in macrophage polarization during liver fibrosis is poorly understood. TRPV4 binds to RhoA via its N-terminal domain (18). The RhoA/ROCK pathway regulates cytoskeletal remodeling, mechanotransduction, and immune cell phenotype switching. ROCK inhibition suppresses M2-like macrophage polarization and relieves experimental fibrosis (19). STAT6 is the main transcription factor driving IL-4-induced M2-associated polarization (20). We speculated that TRPV4 may regulate macrophage phenotype via RhoA/ROCK signaling.

Based on these observations, we hypothesized that CBD alleviates liver fibrosis by targeting TRPV4. We propose that CBD suppresses TRPV4/RhoA/ROCK signaling and STAT6 phosphorylation, thereby inhibiting M2-like macrophage polarization and disrupting the pro-fibrotic crosstalk between macrophages and HSCs. To test this, computational strategies combining network-based pharmacology, docking simulation, and dynamic conformational modeling predicted cannabidiol-TRPV4 molecular binding profiles. We validated these findings in human fibrotic livers, BDL mice, and THP-1-derived macrophages. The role of TRPV4 in macrophage polarization was defined with single-cell transcriptomics, TRPV4 knockdown or overexpression, and pharmacological interventions. Macrophage–LX-2 co-culture was used to assess paracrine effects on HSC activation. This work offers mechanistic insights into cannabidiol-driven antifibrogenic properties and suggests that macrophage-derived TRPV4 signaling may represent a potential therapeutic target for hepatic fibrogenesis.

## 2 Materials and methods

### 2.1 Reagents and antibodies

All drugs, reagents, and cell lines utilized in this study were sourced as follows: Cannabidiol (CBD, No. PHL85705, purity >90%) was acquired from Phyproof (Germany). RN-1734 (No. T7572), GSK1016790A (No. T6848), and silymarin (No. T75437) were procured from TargetMol (USA). The human cell lines of monocytic THP-1 (No. CL2033) and LX-2 (No. CL0560) were obtained from Pricella Biotechnology (Wuhan, China). ELISA kits for TNF-α (No. ml002095) and IL-1β (No. ml106733) were purchased from Enzyme-linked Biotechnology (Shanghai, China). Additional reagents included 5% bovine serum albumin (BSA, No. SW3015), Sirius Red (No. G1472), H&E (No. G1120), Masson’s trichrome (No. G1340), and BCA protein assay kit (No. PC0020) were supplied by Solarbio (Beijing, China). The DAB substrate kit (No. PV-8000) was obtained from ZSGB-BIO (Beijing, China), and the immunoprecipitation kit with protein A/G immunomagnetic beads (No. YJ201) was purchased from Yeasen Biotechnology (Shanghai, China).

Antibodies: Rabbit primary antibodies against Collagen I (1:500, HA722517), ROCK1 (1:2000, ER40122), ROCK2 (1:800, HA722176), RhoA (1:800, ET1611-10), CD68 (1:500, HA722737), CD206 (1:1000, HA722892), iNOS (1:1000, HA722031), GAPDH (1:5000, ET1601-4), and α-SMA (1:5000, ET1607-43) were sourced from Huabio (Zhejiang, China). Rabbit anti-CD163 antibody (1:250, No. A17011) was sourced from Nature Biosciences (Zhejiang, China). Rabbit primary antibodies targeting TRPV4 (1:1000, clone EPR27068-39), STAT6 (1:1000, clone EPR18278-222), Phospho-STAT6 (1:1000, clone EPR22599-78), and TGF-β1 (1:1000, clone EPR21143) were obtained from Abcam (UK). Rabbit anti-IL-10 antibody (1:1000, No. CY6705) was purchased from ABWAYS (Sichuan, China). Recombinant Goat Anti-Rabbit IgG (H+L) Secondary Antibody (1:300, RGAR003) was supplied by Proteintech (Wuhan, China). Flow cytometry antibodies, including CD45 PerCP-Cy5.5, F4/80 FITC, and CD86 APC, were acquired from MultiSciences (China), and TruStain FcX™ PLUS was from eBioscience. Additionally, recombinant IL-4 (No. AF21414) from PeproTech (Hamburg, Germany), and phorbol 12-myristate 13-acetate (PMA, No. P8139) from Sigma (USA).

### 2.2 Animal procedures and treatments

Male C57BL/6J mice (6–8 weeks old, 20 ± 2 g) were purchased from Tianqin Biotechnology (Changsha, China). Animals were acclimatized in standardized housing (55–60% humidity, 22–24 °C, 12-hour light-dark cycle) for 1 week before experiments. All *in-vivo* operations followed the NIH Guide for the Care and Use of Laboratory Animals (NIH Publication No. 80-23, revised 1978) and gained ethical authorization from the Animal Experimental Ethical Inspection Committee of Guizhou Medical University (Approval No. 2304864). Humane care was implemented throughout the study.

To establish the BDL-induced hepatic fibrosis model, mice received i.p. 1% pentobarbital sodium (50 mg/kg) for anesthesia. The common bile duct was surgically exposed and doubly ligated using 6–0 silk sutures; sham-operated mice received identical laparotomy procedures without bile duct ligation. Postoperatively, mice were randomized into experimental groups (n = 6–8 per group). Beginning two weeks after BDL surgery, mice received daily intraperitoneal delivery of phosphate-buffered saline (PBS, vehicle control), cannabidiol (CBD, 4 or 8 mg/kg) (21), RN-1734 (30 mg/kg) (22), or combined CBD-RN-1734 treatment for 7 consecutive days. Silymarin (50 mg/kg) was administered as a positive control. The experimental protocols are illustrated in **Figure 1A**.

**Figure 1:**
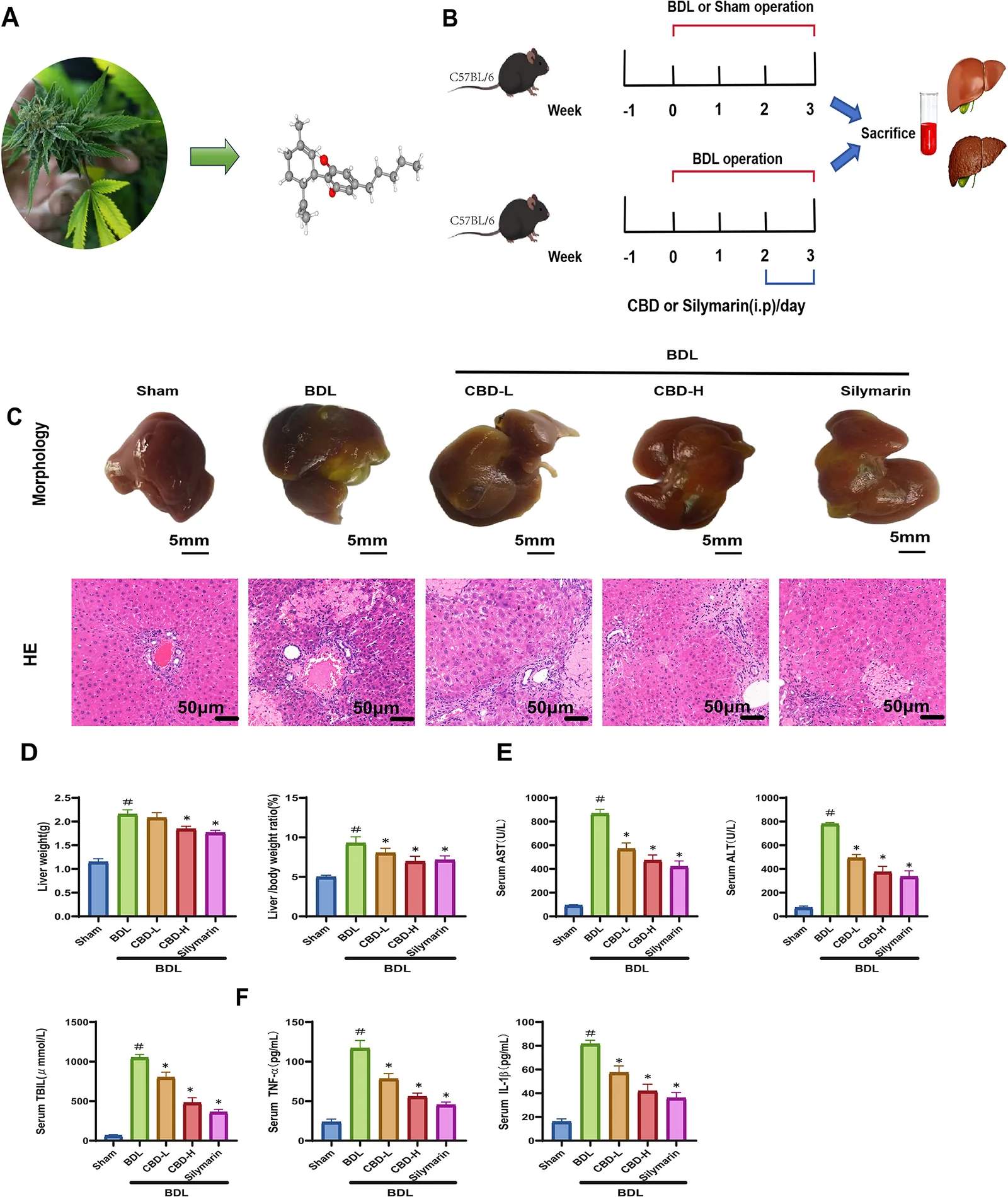
Cannabidiol mitigates bile duct ligation-provoked hepatic injury and inflammatory cascades in mice. **(A)** Photograph of the Cannabis sativa plant alongside the two-dimensional chemical structure of cannabidiol (CBD). **(B)** Schematic overview of the *in vivo* murine intervention regimen. Study cohorts included sham-operated controls, bile duct-ligated (BDL) mice, low-dose CBD-treated BDL mice (4 mg/kg), high-dose CBD-treated BDL mice (8 mg/kg), and silymarin-positive control BDL mice (50 mg/kg). **(C)** Macroscopic hepatic appearances and corresponding H&E-stained histological sections from each cohort (scale bars: 5 mm for gross views, 50 μm for micrographs). **(D)** Quantitative assessment of the hepatosomatic index (hepatic weight relative to total body weight) in all study groups (n = 6–8 per cohort). **(E)** Circulating levels of hepatic function biomarkers TBIL, ALT, and AST in response to BDL-mediated liver insult (n = 6–8). **(F)** Quantification of serum pro-inflammatory mediators TNF-α and IL-1β via ELISA detection (n = 6–8). Data are expressed as mean ± SEM (n = 6–8 per group).*^#^P* < 0.05 relative to the Sham cohort; ^*^P < 0.05 relative to the BDL cohort.

### 2.3 Human liver specimens

Specimens of human hepatic tissue were obtained via the Affiliated Hospital of Guizhou Medical University after receiving signed consent from recruited patients. Fibrotic liver tissues (n = 6) were harvested from patients undergoing liver transplantation or resection for hepatocellular carcinoma, whereas non-fibrotic normal hepatic samples (n = 6) were isolated from histologically unaffected regions adjacent to hepatic hemangioma lesions. Pathological information was supplied by the Department of Pathology. Ethics Committee approval was secured (Approval No. 2023, Ethics Review No. 454). The available clinical characteristics of the patients, including age, sex, etiology, fibrosis stage, and liver function, are summarized in **Supplementary Table 2**.

### 2.4 Isolation and flow cytometric analysis of hepatic macrophages

Sequential differential pelleting enriched hepatic non-parenchymal populations. Low-speed sedimentation at 50 × g for 5 min depleted hepatocytes, followed by pelleting at 800 × g for 5 min. The pellet was resuspended in red blood cell lysis buffer, incubated at 4 °C for 10–15 min, and washed with PBS containing 5% FBS. Hepatic macrophages were purified by density gradient centrifugation using 20% Nycodenz (500×g, 15 min, 4 °C). The macrophage-containing layer was collected and washed.

For flow cytometric profiling, freshly isolated hepatic macrophages were pre-incubated with Fc blocking reagent at ambient temperature for 10 min to eliminate non-specific antibody binding. Surface immunolabeling was subsequently performed using fluorophore-tagged antibodies targeting PerCP-Cy5.5-CD45, FITC-F4/80, and APC-CD86, with incubation carried out at 4 °C for 30 min under light-shielded conditions. For intracellular detection of CD206, fixed cells were permeabilized with BD-supplied Permeabilization Buffer prior to staining with PE-conjugated anti-CD206 antibody. Flow cytometric data were captured using a BD FACS Canto platform and further processed via FlowJo software (version 10.6.2). The gating scheme for defining macrophage subsets (CD45^+^F4/80^+^CD86^+^ for M1-polarized macrophages and CD45^+^F4/80^+^CD206^+^ for M2-polarized counterparts) is detailed within the **supplementary materials**.

### 2.5 Immunohistochemistry

Heat-induced epitope retrieval was performed on hepatic tissue sections using sodium citrate buffer. After a cooling interval, tissue slices underwent thorough rinsing and were pre-blocked with 5% goat serum to reduce off-target antibody binding, followed by overnight incubation with primary antibodies at 4 °C. Post-washing steps were completed before incubation with secondary antibodies for 1 h. Chromogenic visualization was achieved via diaminobenzidine (DAB) staining, while 10% hematoxylin was employed for nuclear counterstaining. Microscopic images of stained sections were acquired using an optical microscope.

### 2.6 Cell culture, polarization, co-culture, and flow cytometry

THP-1 monocytic cells were differentiated into unpolarized M0 macrophages via 24-h stimulation with 150 nM phorbol-12-myristate-13-acetate (PMA), followed by a subsequent 24-h resting period. Pro-inflammatory M1-like macrophage polarization was triggered by 48-h exposure to 100 ng/mL lipopolysaccharide (LPS), whereas alternative M2-associated polarization was induced using 30 ng/mL recombinant interleukin-4 (IL-4) over the same duration. A CCK-8 assay was utilized to screen cell viability following 12- or 24-h compound administration, with non-cytotoxic concentrations determined as 5 μM for cannabidiol (CBD), 0.5 μM for GSK1016790A, and 5 μM for RN-1734. Apoptotic events were quantified with the Annexin V-FITC apoptosis detection kit after 24-h intervention with 5 μM CBD. For flow cytometric detection, CBD-treated polarized THP-1-derived macrophages were immunolabeled with PE-coupled anti-CD206 (phenotypic marker) or APC-conjugated anti-CD86 (M1 phenotypic marker) at 4 °C for 30 min, and data were acquired on a BD FACS Canto cytometer and processed using FlowJo software. Conditioned medium (CM) derived from M2-like macrophages post-24-h serum-free culture and compound treatment was harvested, subjected to pelleting, diluted 1:1 with fresh DMEM medium, and further incubated with LX-2 hepatic stellate cells for 48 h to evaluate cell migratory capacity and profibrogenic activation status.

### 2.7 Bioinformatics analyses

To identify CBD targets, TCMSP (44 candidates) and fibrosis genes from TTD/OMIM/GeneCards were intersected. The interaction networks between proteins were evaluated via combined application of STRING and Cytoscape. Molecular docking with AutoDock Vina predicted CBD-TRPV4 binding (ΔG). Molecular dynamics simulation (Amber24, 250 ns) assessed complex stability via RMSD, Rg, SASA, RMSF, and hydrogen bonds.

### 2.8 TRPV4 profiling via single-cell RNA-seq

ScRNA-seq datasets from normal and fibrotic mouse livers were obtained from a public database (GSE136103). Cell annotation, TRPV4 expression profiling, macrophage subpopulation analysis, and correlation with functional genes were performed to evaluate TRPV4 expression characteristics in macrophages and its association with M2-associated polarization.

### 2.9 Lentiviral transfection and generation of stable cell lines

Lentiviral constructs for TRPV4 overexpression, TRPV4-targeted knockdown, and corresponding negative control vectors were generated by Beijing Duona Biotechnology Co., Ltd. (Beijing, China). The overexpression backbone adopted the plenti-CMV-MCS-EF1a-ZsGreen-T2A-Puro plasmid, whereas the shRNA-mediated knockdown vector was plvx-shRNA-CMV-ZsGreen1-T2A-Puro. THP-1 monocytes were seeded at a density of 5 × 10^4^ cells per well and subjected to lentiviral transduction at a multiplicity of infection (MOI) of 50, with 100 μg/mL transduction enhancer supplemented during infection. Following a 12-h incubation period, the culture medium was refreshed, and cells were maintained for an additional 72 h; EGFP expression was subsequently verified by fluorescence microscopy. Stable transgenic cell populations were screened using 3.5 μg/mL puromycin over 2–3 consecutive passages. The efficacy of TRPV4 overexpression or silencing was assessed by qRT-PCR and WB. The established stable cell lines were expanded and cryopreserved for subsequent experiments.

### 2.10 Co-immunoprecipitation and mass spectrometry analysis

THP-1-derived macrophages were lysed after IL-4 stimulation. The lysates were incubated with anti-TRPV4 or anti-RhoA antibodies and magnetic beads overnight, and the eluates were analyzed by Western blot. Separate lysates were incubated with anti-TRPV4 antibody, and the bead-enriched complexes were subjected to mass spectrometry identification.

### 2.11 Statistical analysis

All statistical analyses were conducted using GraphPad Prism 8.0 software (GraphPad Software, USA). Quantitative data are expressed as mean ± standard error of the mean (SEM) unless otherwise specified. All *in vitro* and *in vivo* experiments were independently replicated a minimum of three times. An unpaired Student’s t-test was employed to assess differences between the two experimental groups. For comparisons across multiple experimental cohorts, one-way analysis of variance (ANOVA) was applied, followed by Tukey’s *post-hoc* test for pairwise *post-hoc* comparisons. A P-value of less than 0.05 was considered to indicate a statistically significant difference.

## 3 Results

### 3.1 CBD alleviates BDL-induced liver injury and inflammation

To delineate the *in-vivo* hepatoprotective and anti-inflammatory effects of CBD, a murine hepatic fibrosis model was established via BDL surgery. The animal experimental workflow is schematized in **Figure 1B**. CBD intervention mitigated BDL-triggered hepatic pathological lesions, including parenchymal necrosis and inflammatory cell infiltration (**Figure 1C**). The increased liver-to-body weight ratio in BDL-model mice was partially normalized following CBD administration (**Figure 1D**). BDL insult markedly elevated serum concentrations of ALT, aspartate AST, and TBIL, core hepatic function biomarkers, whereas CBD dose-dependently reversed these pathological perturbations (**Figure 1E**). ELISA quantification further demonstrated that CBD lowered circulating pro-inflammatory IL-1β and TNF-α levels in BDL-operated mice (**Figure 1F**). Cumulatively, our observations verify that CBD exerts partial but significant protective effects against BDL-induced hepatic injury and inflammatory cascades in fibrotic murine livers.

### 3.2 CBD attenuates hepatic fibrosis progression in BDL-treated mice

Histological analysis demonstrated that CBD dose-dependently reduced collagen fiber deposition in hepatic tissue sections (**Figure 2A**). IHC staining revealed that BDL surgery induced a marked upregulation of α-SMA and Col-I expression in mouse liver tissues, which was significantly attenuated by CBD treatment (**Figure 2B**). Consistent with the histological observations, immunoblotting confirmed that CBD effectively mitigated the BDL-induced upregulation of α-SMA and Col-I (**Figure 2C**). These results collectively indicate that CBD ameliorates BDL-induced hepatic fibrogenesis.

**Figure 2:**
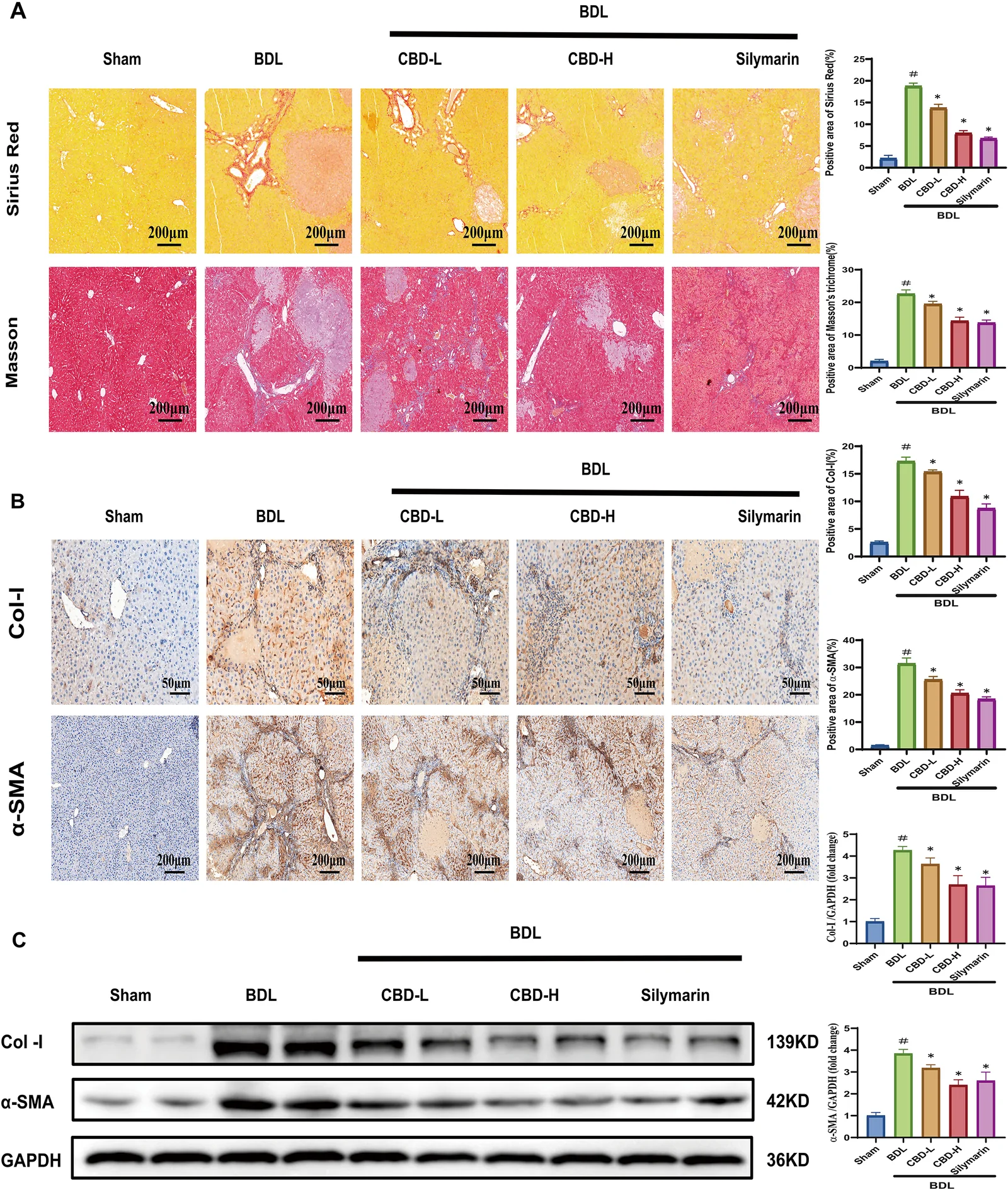
Cannabidiol hinders progressive hepatic fibrogenesis in bile-duct-ligated murine models. Post-BDL surgical manipulation, experimental mice were intraperitoneally injected with CBD or silymarin, and hepatic specimens were isolated for subsequent pathological and molecular assessment. **(A)** Extracellular collagen buildup was visualized through Masson’s trichrome and Sirius Red histological staining. **(B)** Immunohistochemical profiling characterized the expression signatures of fibrotic biomarkers Col-I and α-SMA. **(C)** Immunoblotting was adopted to quantify the relative protein abundances of α-SMA and Col-I within hepatic samples. Data are expressed as mean ± SEM (n = 6–8 per cohort).^#^*P <* 0.05 relative to the Sham cohort; ^*^P < 0.05 relative to the BDL cohort.

### 3.3 CBD reduces M2-like macrophage infiltration and polarization in BDL-induced Liver fibrosis

Macrophage-mediated inflammation is a well-recognized driver of hepatic fibrogenesis, particularly through the expansion of profibrotic M2-polarized macrophages. To characterize hepatic macrophage subsets, immunofluorescence staining was performed using F4/80 (pan-macrophage marker), CD206 (M2-associated marker), and inducible nitric oxide synthase (iNOS, M1-associated marker). Compared with the sham-operated group, BDL mice exhibited significantly increased numbers of F4/80^+^, CD206^+^ (M2), and iNOS^+^ (M1) cells in liver sections. CBD treatment dose-dependently reduced the infiltration of all macrophage populations, with a more pronounced effect on M2-like macrophages (**Figure 3A**).

**Figure 3:**
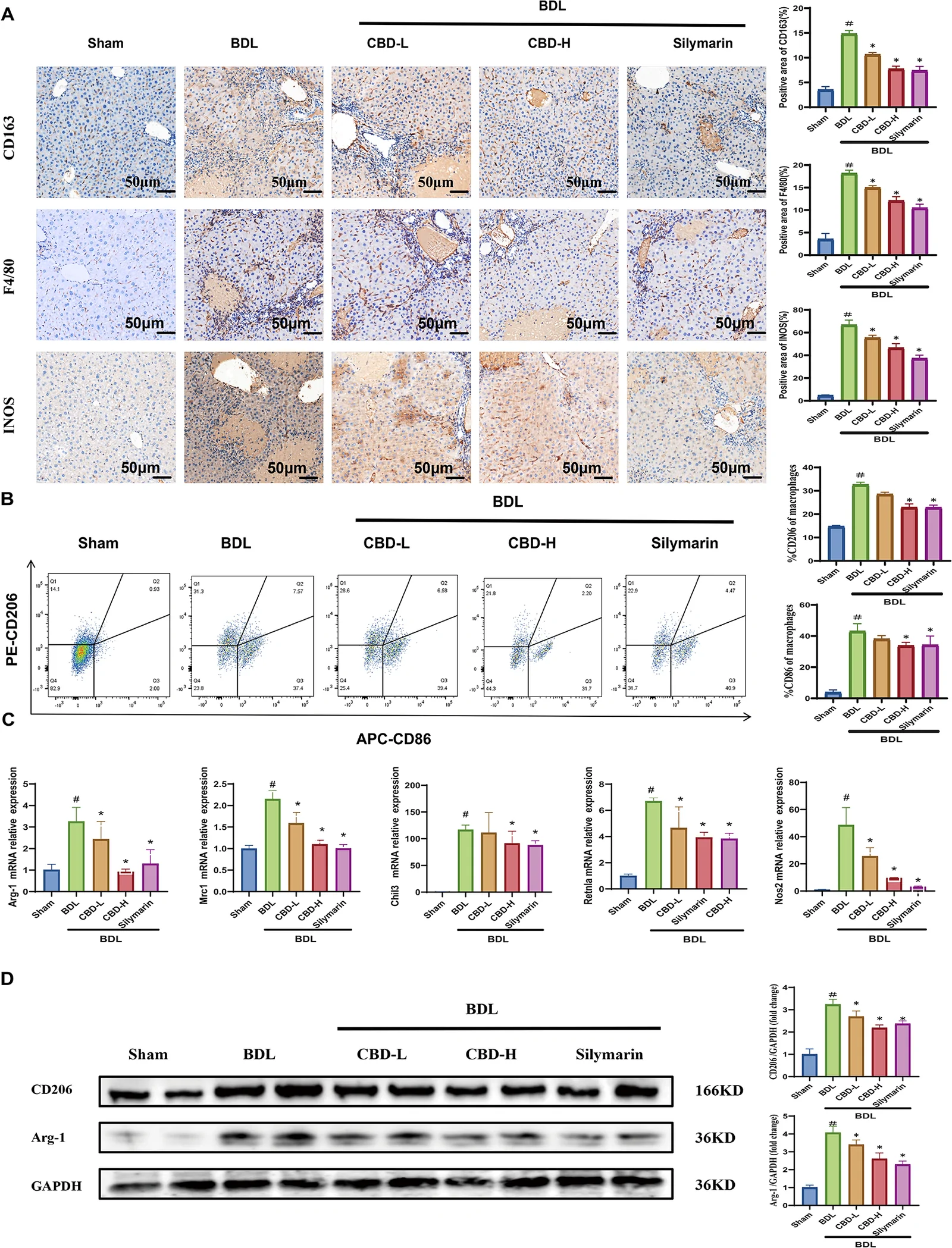
Cannabidiol inhibits hepatic macrophage infiltration and M2-skewed polarization in bile-duct-ligated murine models. **(A)** Immunohistochemical visualization and morphometric quantification of canonical M1/M2-like macrophage biomarkers (CD163, F4/80, iNOS) within hepatic specimens (scale bar = 50 μm). **(B)** Cytometric sorting and phenotyping of hepatic-derived M2-like (CD206^+^CD86^-^) and M1-like (CD206^-^CD86^+^) macrophage subpopulations. **(C)** Quantitative reverse-transcription PCR (RT-qPCR) detection of transcript abundances for M2-like polarization-linked functional genes (Arg-1, Mrc1, CD163, Retnla, Chil3). **(D)** Western immunoblot assays combined with densitometry-based quantification of CD206 and Arg-1 protein levels, with GAPDH set as the housekeeping reference standard. Data are expressed as mean ± SEM (n = 6–8 per cohort). *^#^P* < 0.05 relative to the Sham cohort; ^*^P < 0.05 relative to the BDL cohort.

To further quantify the proportions of M1-like and M2-like macrophages, flow cytometry was performed. Total hepatic macrophages were gated as CD45^+^F4/80^+^ cells, with M2-like macrophages defined as CD45^+^F4/80^+^CD206^+^ and M1-like macrophages as CD45^+^F4/80^+^CD86^+^. BDL challenge resulted in marked expansions of both CD206^+^ (M2) and CD86^+^ (M1) cell populations; CBD treatment significantly decreased these populations, with a preferential inhibitory effect on M2-like macrophages (**Figure 3B**).

qRT-PCR analysis of macrophage polarization-related genes showed that BDL significantly elevated the mRNA levels of M2-associated genes (Arg1, Retnla, Mrc1, Chil3) and the M1-associated gene Nos2. CBD treatment effectively reversed these transcriptional changes (**Figure 3C**). Consistently, BDL increased the mRNA levels of inflammatory and profibrotic mediators (Nos2, Tnf-α, Il-10, and Tgf-β), whereas CBD dose-dependently decreased them, with the greatest reductions in the high-dose CBD and silymarin groups (**Supplementary Figure 1A**). Western blot analysis further confirmed that CBD dose-dependently reduced the protein expression of M2-associated markers CD206 and Arg1 in liver tissues (**Figure 3D**). Taken together, these results demonstrate that CBD effectively suppresses hepatic macrophage recruitment and preferentially inhibits M2-like polarization in BDL-induced liver fibrosis.

To further explore macrophage heterogeneity beyond M1/M2 markers, we examined Trem2, Cd9, and Spp1 expression by RT-qPCR. BDL injury significantly upregulated all three markers, and CBD treatment dose-dependently reduced their expression (*P* < 0.05; **Supplementary Figure 1A**). These results suggest that CBD attenuates a broader pro-fibrotic macrophage program beyond classical M2-associated markers.

### 3.4 Identification of TRPV4 as a candidate target of CBD in hepatic fibrosis

To identify potential molecular targets of CBD in liver fibrosis, we intersected CBD-related genes with liver fibrosis-associated genes retrieved from public databases, yielding 32 overlapping candidate genes (**Figure 4A**). PPI network constructed using the STRING database identified transient receptor potential vanilloid 4 (TRPV4) as a hub gene (**Figure 4B**). KEGG pathway overrepresentation analysis revealed that TRP channels were among the top-ranked signaling pathways (**Figure 4C**), prompting us to select TRPV4 for further mechanistic investigation. Furthermore, GO enrichment analysis showed that these genes were significantly enriched in plasma membrane components, metal ion transport, transmembrane signaling receptor activity, and calcium channel activity (**Figure 4D**). Notably, the enrichment of calcium channel activity further supports TRPV4 as a key target, prompting us to select TRPV4 for further mechanistic investigation.

**Figure 4:**
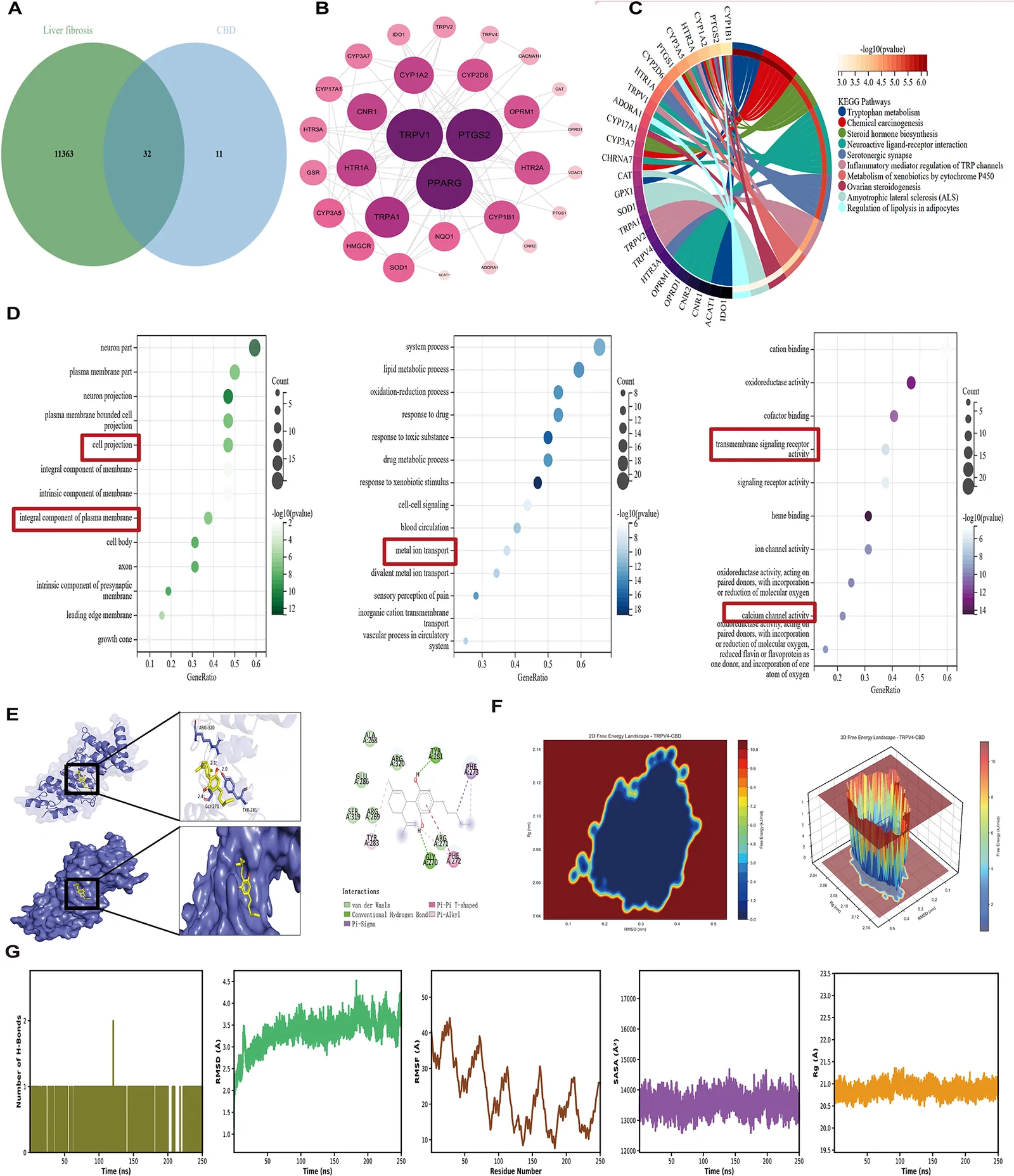
Identification of potential CBD-targeted molecules underlying hepatic fibrogenesis **(A)** Venn diagram showing the overlap between genes implicated in liver fibrosis and targets that bind CBD. **(B)** PPI network of overlapping targets. **(C)** GO enrichment analysis of intersecting genes. **(D)** KEGG pathway enrichment analysis of intersecting genes; **(E)** Molecular docking analysis of CBD with TRPV4. **(F)** Free energy landscape analysis. **(G)** Assessment of hydrogen bond number, RMSD, SASA, and Rg for the CBD–TRPV4 complex.

Molecular docking analysis demonstrated that CBD forms three hydrogen bonds with TRPV4 residues ARG-320, GLY-270, and TYR-281 (distance to TYR-281: 2.0 Å), as well as five π-stacking interactions with TYR-283, PHE-272, and PHE-273, indicating a binding affinity (**Figure 4E**).

To assess intermolecular binding stability, 250-ns molecular dynamics (MD) simulations were conducted (**Figure 4G**). Quantitative profiling of intermolecular hydrogen-bond interactions demonstrated that the total count of hydrogen-bond contacts at the CBD-TRPV4 binding interface remained within 0–1 over the entire simulation period, with only minor temporal oscillations, confirming robust hydrophilic intermolecular association. Conformational stability analysis showed that the root-mean-square deviation (RMSD) curve of the complex remained within 2 Å with no significant drift. The radius of gyration (Rg) curve was stable, with only a slight fluctuation around 60 ns, consistent with a transient increase in solvent-accessible surface area (SASA) at that time point, reflecting good structural compactness. Root-mean-square fluctuation (RMSF) analysis demonstrated low mobility of key residues in the binding interface (e.g., ILE-331), further supporting conformational stability. The binding free energy (ΔG_binding) calculated by the MM/GBSA method was −5.8 kcal/mol. The free energy landscape (**Figure 4F**), constructed as a two-dimensional RMSD-Rg plot, identified stable conformational clusters of the CBD-TRPV4 complex in the region of low RMSD (<2 Å) and moderate Rg, corresponding to a deep-blue energy valley. Collectively, these multi-scale computational analyses demonstrate that CBD forms a structurally stable and computationally stable binding pose with TRPV4, providing a structural dynamics basis for its antifibrotic effects. Fluo-4 AM calcium imaging in THP-1-derived macrophages showed that GSK1016790A-induced Ca²^+^ influx was significantly attenuated by CBD pretreatment, providing functional evidence that CBD may inhibit TRPV4 channel activity (**Supplementary Figure S4**).

### 3.5 Single-cell transcriptomics reveals preferential TRPV4 expression in macrophages during liver fibrosis

We analyzed a public single-cell RNA-sequencing (scRNA-seq) dataset (GSE136103) from healthy and fibrotic mouse livers to quantify cell-type-specific TRPV4 expression (**Figure 5A**). Overall, TRPV4 expression was sparse (3.2% of 12,847 cells) and distributed across diverse cell types (**Figure 5B**). However, it exhibited pronounced cell-type selectivity: the highest expression levels were observed in macrophages, with Kupffer cells showing a 5.37% positive rate (49/912; mean expression 0.060), followed by lipid-associated macrophages (4.35%, 32/735; mean 0.045). In contrast, hepatocytes had the lowest TRPV4 expression (2.15%, 125/5832; mean 0.018) (**Figure 5C**). Consistently, UMAP and heatmap analyses (**Supplementary Figures 2A, B**) revealed increased TRPV4 expression in macrophages from fibrotic livers, particularly in Kupffer cells and lipid-associated macrophages.

**Figure 5:**
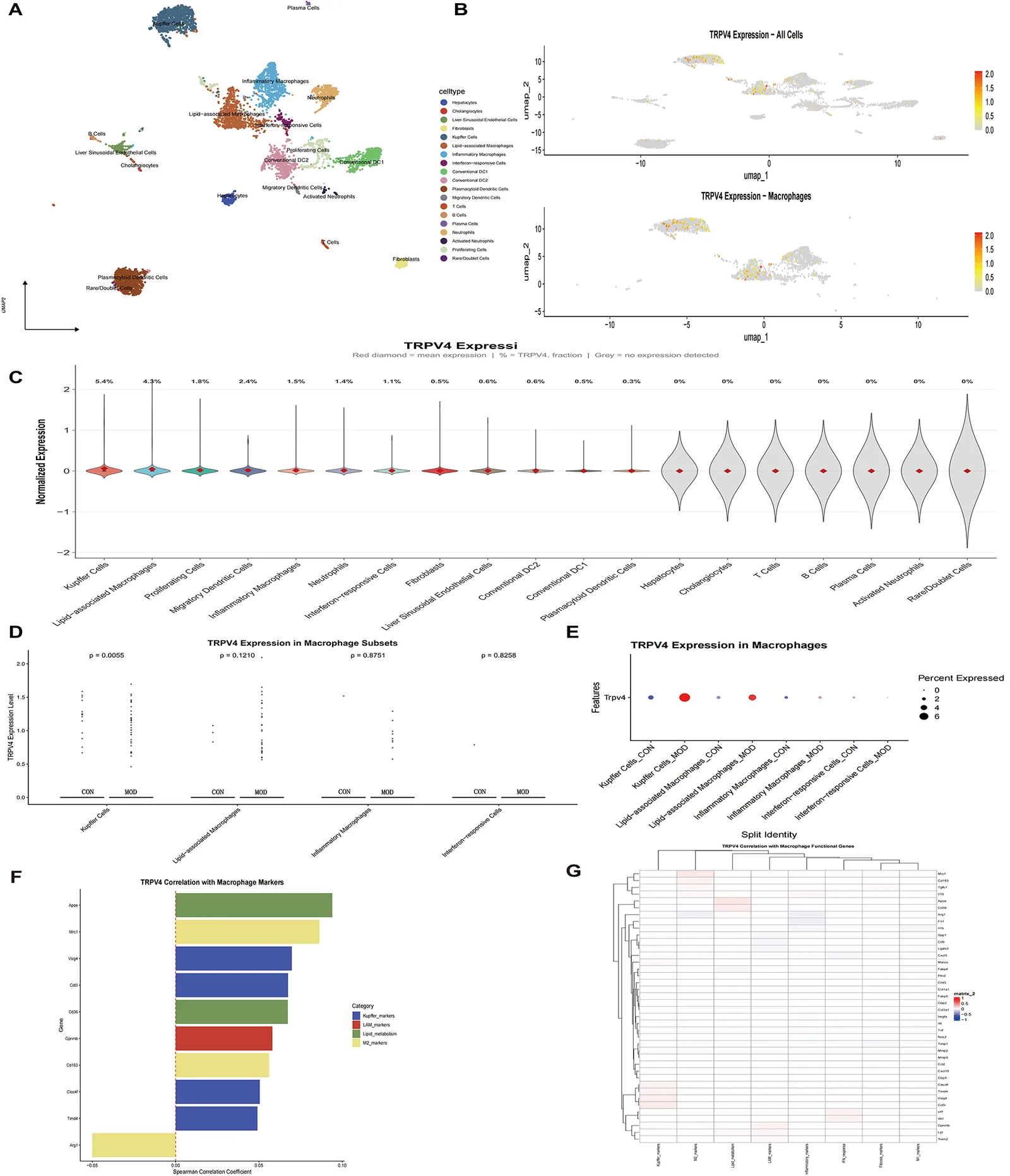
Single-cell RNA-seq atlas identifies cell-type-specific TRPV4 expression in MOD fibrotic mouse livers relative to CON controls. **(A)** UMAP plot. **(B)** Distribution of TRPV4 expression in the UMAP plot. **(C)** Density plot of Trpv4 expression. **(D)** Boxplot illustrating TRPV4 signature scores in macrophages. **(E)** Macrophage annotation and corresponding dot plot of Trpv4 expression. **(F)** Bar plot depicting correlations between Trpv4 and M2-associated marker genes. **(G)** Heatmap illustrating correlations between Trpv4 and M1 marker genes.

Among macrophage subsets, Kupffer cells harbored the maximal TRPV4-positive proportion (7.40%) and mean expression intensity (0.080), with these metrics statistically augmented in fibrotic liver tissues versus healthy hepatic counterparts (*P* = 0.0055) (**Figure 5D**). Dot plot and heatmap analyses further confirmed the enrichment of TRPV4 in Kupffer cells (**Figure 5E**).

Spearman’s rank-order correlation demonstrated that TRPV4 expression positively covaried with.

M2-associated genes (Mrc1, r = 0.086; Cd163, r = 0.056) and lipid metabolism-related genes (Apoe, r = 0.094; Cd36, r = 0.067), but not with M1 marker genes (Il-1b, Tnf-α, Nos2) (**Figures 5F, G**). These findings indicate that TRPV4 is primarily enriched in macrophages, particularly Kupffer cells, and may be involved in M2-like polarization and lipid metabolism rather than acute inflammatory responses. Bulk RNA-seq analysis of human normal and fibrotic liver tissues further supports the clinical relevance of TRPV4-associated signaling in liver fibrosis (**Supplementary Figure 3**).

The above findings demonstrate that TRPV4 is highly expressed in macrophages, particularly Kupffer cells, and correlates with M2-like polarization during liver fibrosis. However, the downstream signaling mechanisms through which TRPV4 contributes to fibrogenesis remain unclear. Notably, TRPV4 is a direct binding partner of Ras homolog family member A (RhoA) and activates its downstream effector Rho-associated coiled-coil containing protein kinase (ROCK). This signaling axis is known to regulate HSCs activation, extracellular matrix deposition, and fibrotic progression. Given the critical crosstalk between macrophages and HSCs in liver fibrosis, we hypothesized that TRPV4 promotes fibrosis via the RhoA/ROCK pathway. To test this hypothesis, we next examined whether CBD modulates the TRPV4/RhoA/ROCK signaling axis in the fibrotic liver.

### 3.6 CBD inhibits the TRPV4/RhoA/ROCK signaling axis in BDL mouse livers

Immunohistochemical staining for TRPV4 revealed that compared with the sham group, BDL mice showed a marked increase in TRPV4-positive cells, which were predominantly localized in non-parenchymal cells. CBD treatment dose-dependently reduced the number of TRPV4-positive cells (**Figure 6A**).

**Figure 6:**
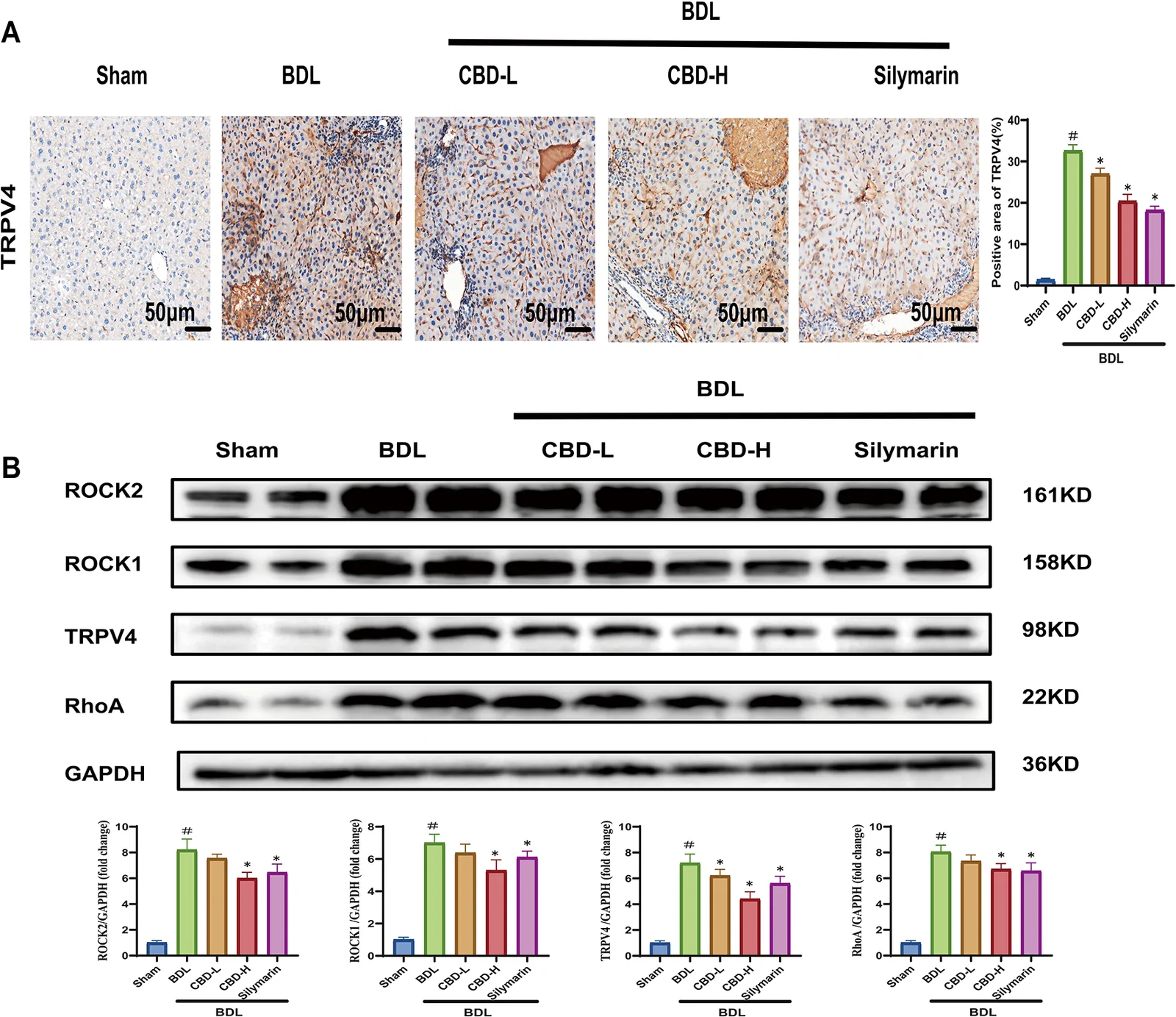
CBD blocks the TRPV4/RhoA/ROCK signaling cascade in the liver of BDL-operated mice. **(A)** IHC staining assessment of TRPV4 protein expression in hepatic tissues. **(B)** Immunoblotting quantification of TRPV4, RhoA, ROCK1, and ROCK2 protein abundances in liver specimens. Data are presented as mean ± SEM (n = 6 per group).*^#^P* < 0.05 versus the Sham group; ^*^P < 0.05 versus the BDL group.

Western blot analysis further demonstrated that BDL-induced injury drove robust upregulation of TRPV4-RhoA-ROCK cascade protein expression in hepatic samples. These increases were effectively reversed by CBD treatment in a dose-dependent manner (**Figure 6B**). Overall, these data show that CBD slightly suppresses the TRPV4/RhoA/ROCK signaling axis, suggesting its partial role in inhibiting liver fibrosis.

### 3.7 The TRPV4/RhoA/ROCK pathway is upregulated in human fibrotic liver tissues

Histological examination showed that normal liver tissues displayed intact lobular architecture, whereas fibrotic livers exhibited disrupted structure, marked fibrotic expansion of portal areas, extensive fibrous septa formation, and dense inflammatory cell infiltration. Masson’s trichrome and Sirius Red staining confirmed significantly increased collagen deposition in fibrotic tissues (P < 0.05, **Figure 7A**).

**Figure 7:**
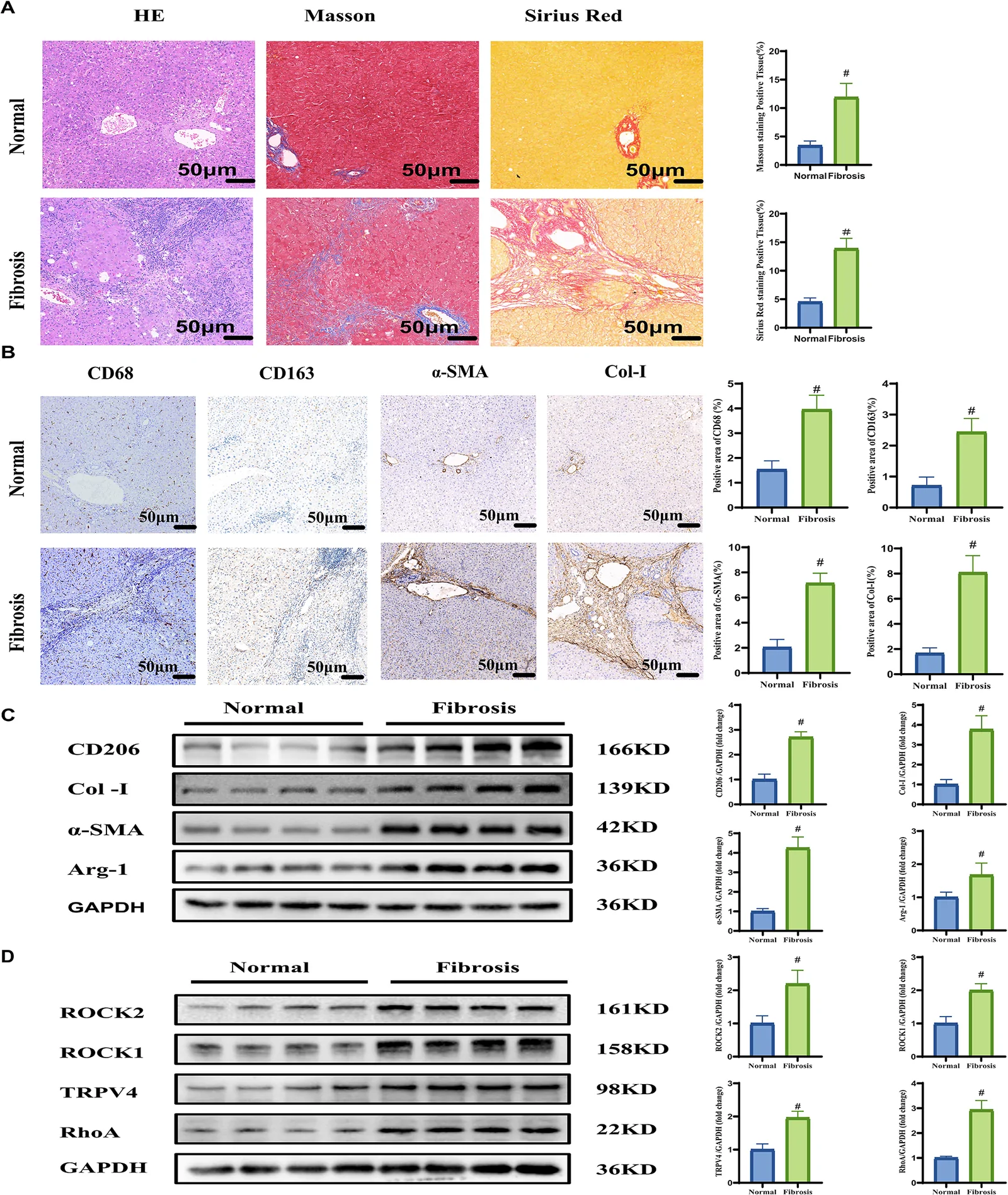
Activation of the TRPV4/RhoA/ROCK pathway and macrophage infiltration in human fibrotic hepatic tissues. **(A)** Representative H&E, Sirius Red, and Masson’s trichrome staining micrographs of normal and fibrotic human liver tissues (scale bar = 50 μm), reflecting pathological collagen deposition and fibrotic severity. **(B)** IHC detection of macrophage (CD68, CD163) and fibrotic (α-SMA, Col-I) marker expression (scale bar = 50 μm). **(C)** Immunoblotting analysis of CD206, Col-I, α-SMA, and Arg-1 protein levels. **(D)** Immunoblotting quantification of TRPV4, RhoA, ROCK1, and ROCK2 protein expression. Data are presented as mean ± SEM (n = 6 per group).*^#^P* < 0.05 versus the Normal group.

Immunohistochemistry revealed that the expression of Col-I and α-SMA, as well as the pan-macrophage marker CD68 and the M2-associated marker CD163, was significantly elevated in fibrotic livers compared with normal controls (*P* < 0.05, **Figure 7B**). Western blot analysis further confirmed the upregulation of Col-I, α-SMA, CD206, and Arg-1 in fibrotic tissues (*P* < 0.05, **Figure 7C**).

Importantly, the protein levels of TRPV4, RhoA, ROCK1, and ROCK2 were also markedly increased in fibrotic livers (*P* < 0.05, **Figure 7D**).The upregulation of these proteins suggests that the TRPV4/RhoA/ROCK axis may represent a promising candidate target for antifibrotic intervention, warranting further functional validation.

### 3.8 CBD may engage TRPV4 and inhibit M2-like macrophage polarization *in vitro*

To characterize CBD-driven modulatory effects on macrophage phenotypic signatures, dose-response profiling was implemented. THP-1-polarized myeloid macrophages received incremental CBD concentrations (1.25–40 μM) following 12- or 24-h treatments (*P* < 0.05).

CCK-8 assays demonstrated that the 2.5–5 μM CBD range exerted no adverse impacts on cellular viability, while 10–40 μM dosages induced time- and dose-dependent cytotoxic effects (*P* < 0.05). Annexin V/PI staining confirmed that 5 μM CBD did not induce significant apoptosis (**Figures 8A, B**). Therefore, 5 μM and lower concentrations (1.25, 2.5 μM) were selected for subsequent experiments.

**Figure 8:**
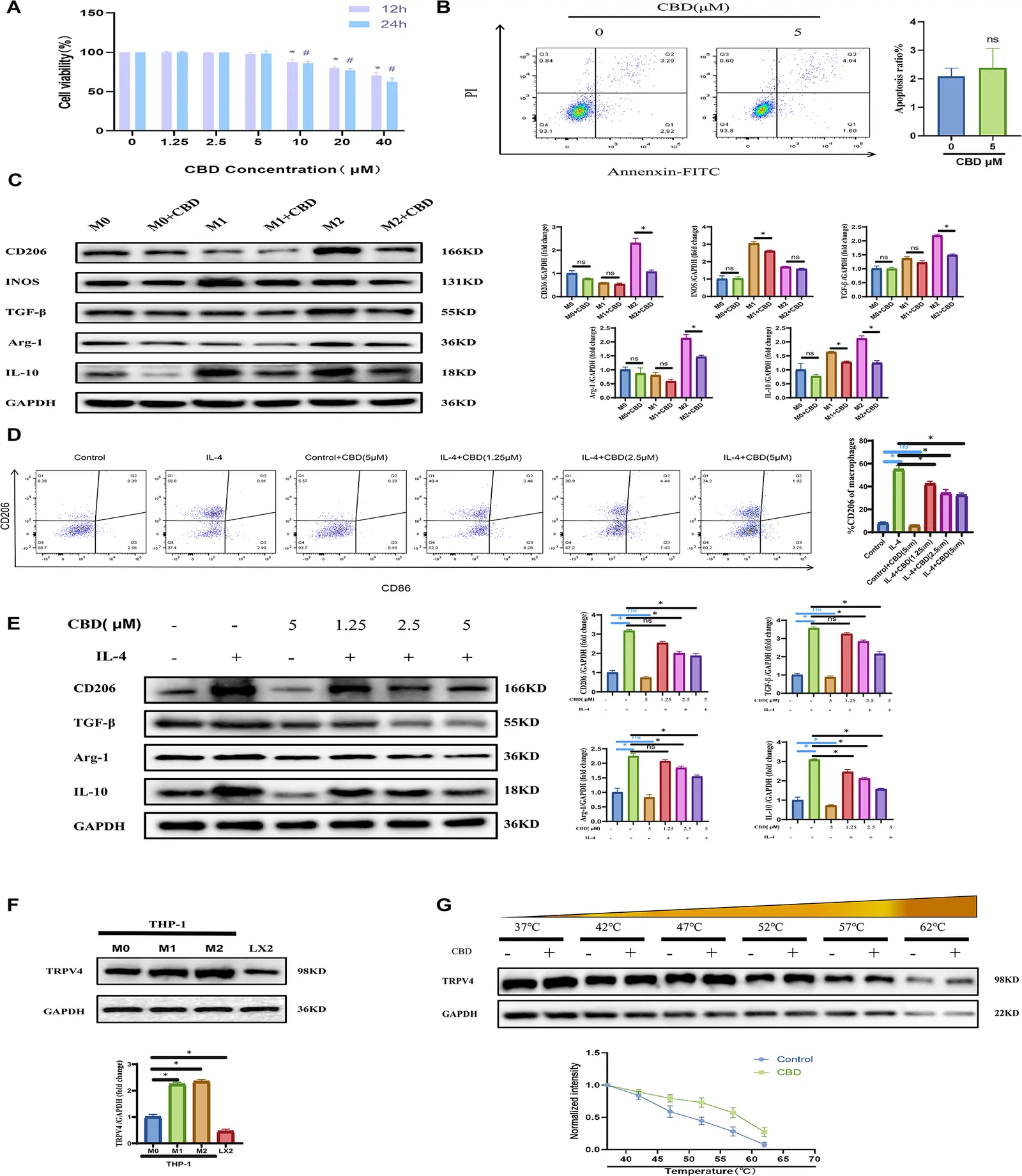
CBD impedes M2-like macrophage polarization via direct TRPV4 binding in THP-1-derived macrophages. **(A)** Relative cell viability of THP-1-differentiated macrophages exposed to gradient concentrations of CBD. **(B)** Flow cytometric detection of apoptosis in THP-1 macrophages with or without 5 μM CBD intervention using Annexin V/PI double staining. **(C)** Immunoblotting measurement of iNOS, CD206, Arg-1, IL-10, and TGF-β protein levels in THP-1 macrophages stimulated with IL-4 (30 ng/ml) or LPS (100 ng/ml), with or without 5 μM CBD treatment. Flow cytometry was applied to quantify CD206^+^ M2-like macrophages following IL-4 stimulation and CBD administration. **(D, E)** Immunoblotting validation of CD206, Arg-1, IL-10, and TGF-β expression in the above cell model. **(F)** Immunoblotting detection of TRPV4 protein expression in THP-1-derived macrophages. **(G)** Cellular thermal shift assay (CETSA) evaluating the thermal stability of the CBD-TRPV4 interaction within a temperature gradient of 37–62 °C. Data are presented as mean ± SEM (n = 3); ^*^P < 0.05; ns, non-significant.

We next examined the effect of CBD on macrophage polarization. LPS-induced M1 polarization markedly upregulated iNOS expression, while IL-4-induced M2-like polarization increased the expression of Arg-1, CD206, transforming growth factor-β (TGF-β), and IL-10. CBD treatment (5 μM) effectively counteracted these polarization-associated changes (**Figure 8C**).

To further evaluate the effect on M2-like polarization, macrophages were treated with IL-4 alone or in combination with CBD at 1.25, 2.5, or 5 μM. Flow cytometric analysis validated that the proportion of CD206^+^ cells was 8.15% in the control group, increasing to 54.15% after IL-4 stimulation. Treatment with 2.5 μM CBD reduced the CD206^+^ population to 43.55%, and 5 μM CBD further decreased it to 34.65% (**Figure 8D**).

Immunoblot profiling corroborated concentration-graduated reductions in CD206, Arg-1, TGF-β, and IL-10 protein abundances upon CBD exposure, with maximal suppression detected at 5 μM (**Figure 8E**). Notably, TRPV4 expression was significantly higher in M2-like macrophages than in M0 or M1 cells, and very low in LX-2 cells (**Figure 8F**). The cellular thermal shift assay (CETSA) demonstrated that CBD enhanced the thermal stability of TRPV4 at temperatures ranging from 47 °C to 62 °C, indicating direct physical binding between CBD and TRPV4 (**Figure 8G**). Collectively, these findings verify that CBD may engage TRPV4 and inhibit M2-like macrophage polarization in a concentration-dependent manner.

### 3.9 TRPV4 knockdown inhibits IL-4-induced M2-like polarization and enhances the inhibitory effect of CBD

To investigate the functional role of TRPV4 in M2-like polarization, a stable TRPV4-knockdown THP-1 cell line (shTRPV4) was generated using shRNA lentivirus, with shControl as a negative control. Western blot and qRT-PCR confirmed that TRPV4 protein and mRNA levels were significantly reduced in shTRPV4 cells compared with shControl cells (*P* < 0.05; **Figure 9A**). Post-macrophage differentiation, IL-4 intervention was applied to elicit M2-type macrophage polarization. Western blot analysis showed that TRPV4 knockdown alone decreased the expression of M2-associated markers CD206, Arg-1, IL-10, and TGF-β (*P* < 0.05). CBD treatment (shControl+CBD) also significantly suppressed these markers, with an effect comparable to that of TRPV4 knockdown. Notably, the combination of shTRPV4 and CBD (shTRPV4+CBD) further reduced the expression of these proteins (*P* < 0.05), supporting a synergistic suppressive action (**Figure 9B**).

**Figure 9:**
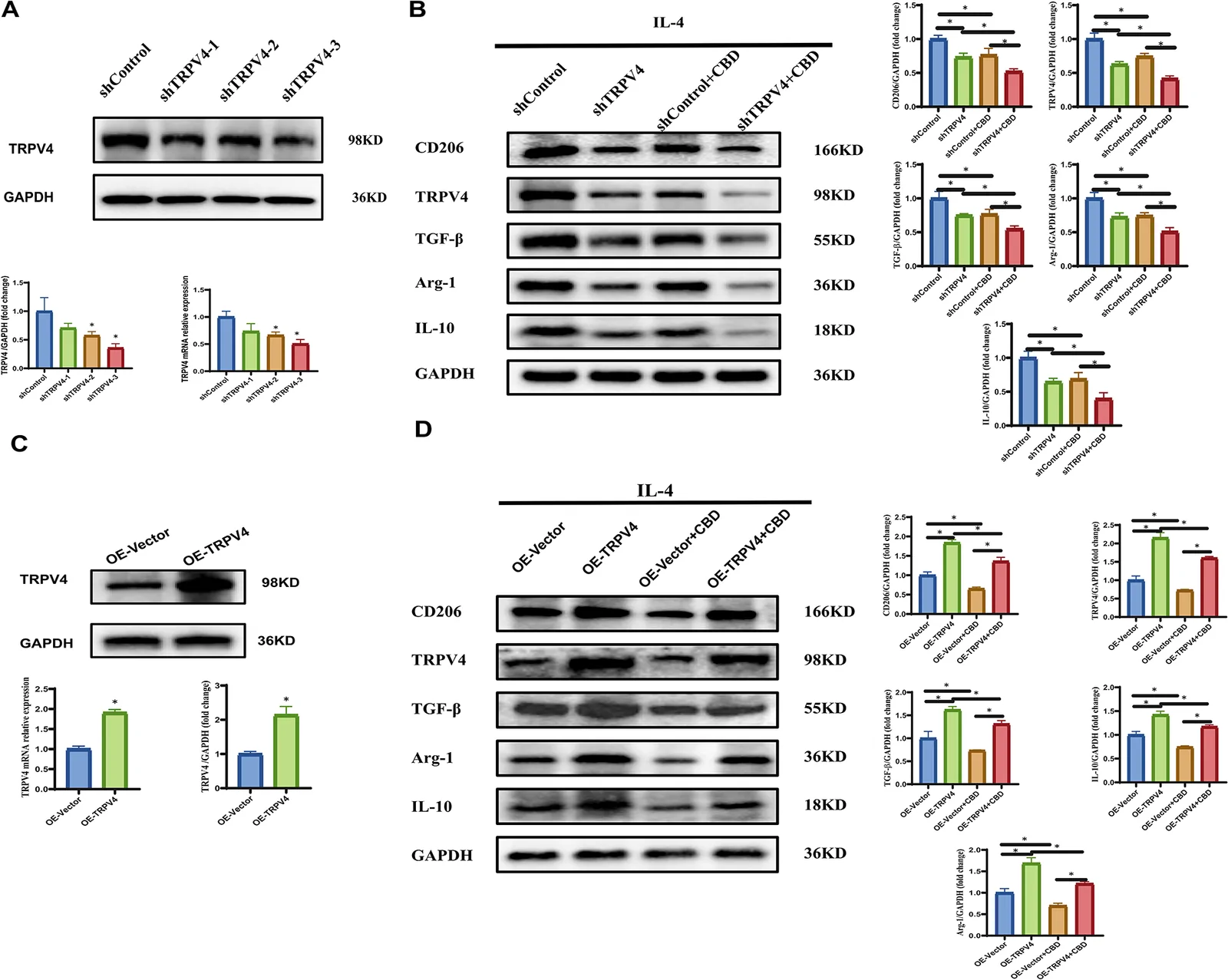
TRPV4 knockdown/overexpression verifies CBD-mediated inhibition of M2-like polarization via TRPV4 in THP-1-derived macrophages. **(A)** Three independent short-hairpin RNA sequences (shTRPV4-1, shTRPV4-2, shTRPV4-3) were constructed to silence TRPV4 expression in THP-1-derived macrophages, with shControl as the negative control. Knockdown efficiency was validated via immunoblotting and qPCR. **(B)** TRPV4-silenced macrophages were stimulated with IL-4 and treated with or without CBD. Immunoblotting was performed to detect TRPV4 and M2-like polarization-related protein (CD206, TGF-β, Arg-1, IL-10) expression. **(C)** TRPV4 overexpression efficiency in THP-1-derived macrophages was confirmed by immunoblotting and qPCR, with an empty OE-Vector as the negative control. **(D)** TRPV4-overexpressing macrophages were subjected to IL-4 stimulation with or without CBD intervention. Immunoblotting analysis quantified TRPV4 and M2-associated marker protein levels. Data are presented as mean ± SEM (n = 3); ^*^P < 0.05; ns, non-significant.

### 3.10 TRPV4 overexpression enhances IL-4-induced M2-like polarization, which is reversed by CBD

A stable TRPV4-overexpressing THP-1 cell line (OE-TRPV4) was established using lentiviral transduction, with OE-Vector as a control. Western blot and qRT-PCR confirmed that TRPV4 protein and mRNA levels were markedly elevated in OE-TRPV4 cells (*P* < 0.05; **Figure 9C**). Upon IL-4 stimulation, OE-TRPV4 cells showed significantly increased protein levels of CD206, Arg-1, IL-10, and TGF-β compared with OE-Vector cells (*P* < 0.05), indicating that TRPV4 overexpression promotes M2-like polarization. Treatment with CBD (OE-TRPV4+CBD) effectively reversed the upregulation of these M2-like markers (*P* < 0.05). Moreover, OE-Vector+CBD also significantly downregulated TRPV4 and M2-associated marker expression (**Figure 9D**). Collectively, these results demonstrate that TRPV4 is a key molecular target through which CBD inhibits M2-like macrophage polarization.

### 3.11 TRPV4 directly interacts with RhoA in macrophages

To clarify the molecular mechanism by which TRPV4 regulates RhoA, Co-IP coupled with mass spectrometry was performed in THP-1-derived macrophages. Mass spectrometry identified multiple potential interaction domains that could mediate binding to RhoA (**Figure 10A**). Under IL-4 stimulation, TRPV4 antibodies co-precipitated RhoA, indicating an endogenous interaction between the two proteins (**Figure 10B**). This result was further confirmed by reverse Co-IP. These findings provide direct molecular evidence that TRPV4 binds to RhoA, supporting that TRPV4 acts upstream of the RhoA/ROCK signaling pathway.

**Figure 10:**
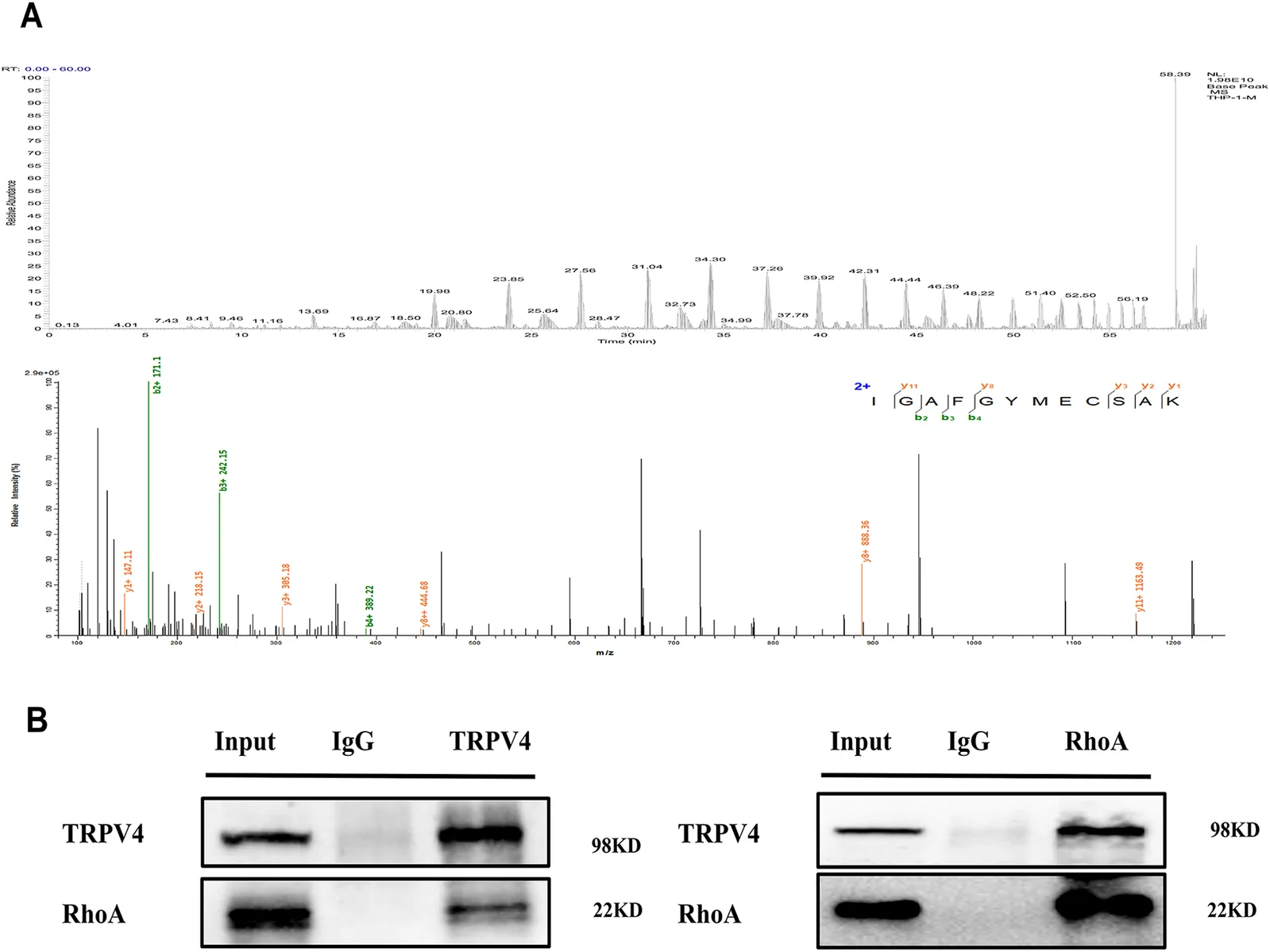
TRPV4 physically interacts with RhoA in THP-1-differentiated macrophages. **(A)** Mass spectrometry screening identified RhoA as a potential TRPV4-binding partner. **(B)** Co-IP assay verified the endogenous physical interaction between TRPV4 and RhoA in THP-1-derived macrophages.

### 3.12 CBD inhibits the TRPV4/RhoA/ROCK signaling axis and STAT6 phosphorylation to suppress M2-like polarization in macrophages

To investigate the TRPV4 downstream pathway in CBD-mediated inhibition of M2-like polarization, the activation status of the RhoA/ROCK pathway and signal transducer and activator of transcription 6 (STAT6) phosphorylation was examined in gain- and loss-of-function models. STAT6 is a master transcription factor that drives M2-like polarization, and its phosphorylation serves as a key readout of M2-like activation.

IL-4 stimulation significantly upregulated the expression of TRPV4, RhoA, ROCK1, ROCK2, and phosphorylated STAT6 (p-STAT6) (*P* < 0.05), confirming activation of the TRPV4/RhoA/ROCK signaling axis and STAT6 phosphorylation during M2-like polarization. CBD treatment dose-dependently suppressed these effects: 1.25 μM CBD showed a decreasing trend without statistical significance; 2.5 μM and 5 μM CBD significantly reduced the levels of RhoA, ROCK1, ROCK2, p-STAT6, and TRPV4 (*P* < 0.05), with the effect observed at 5 μM (**Figure 11A**).

**Figure 11:**
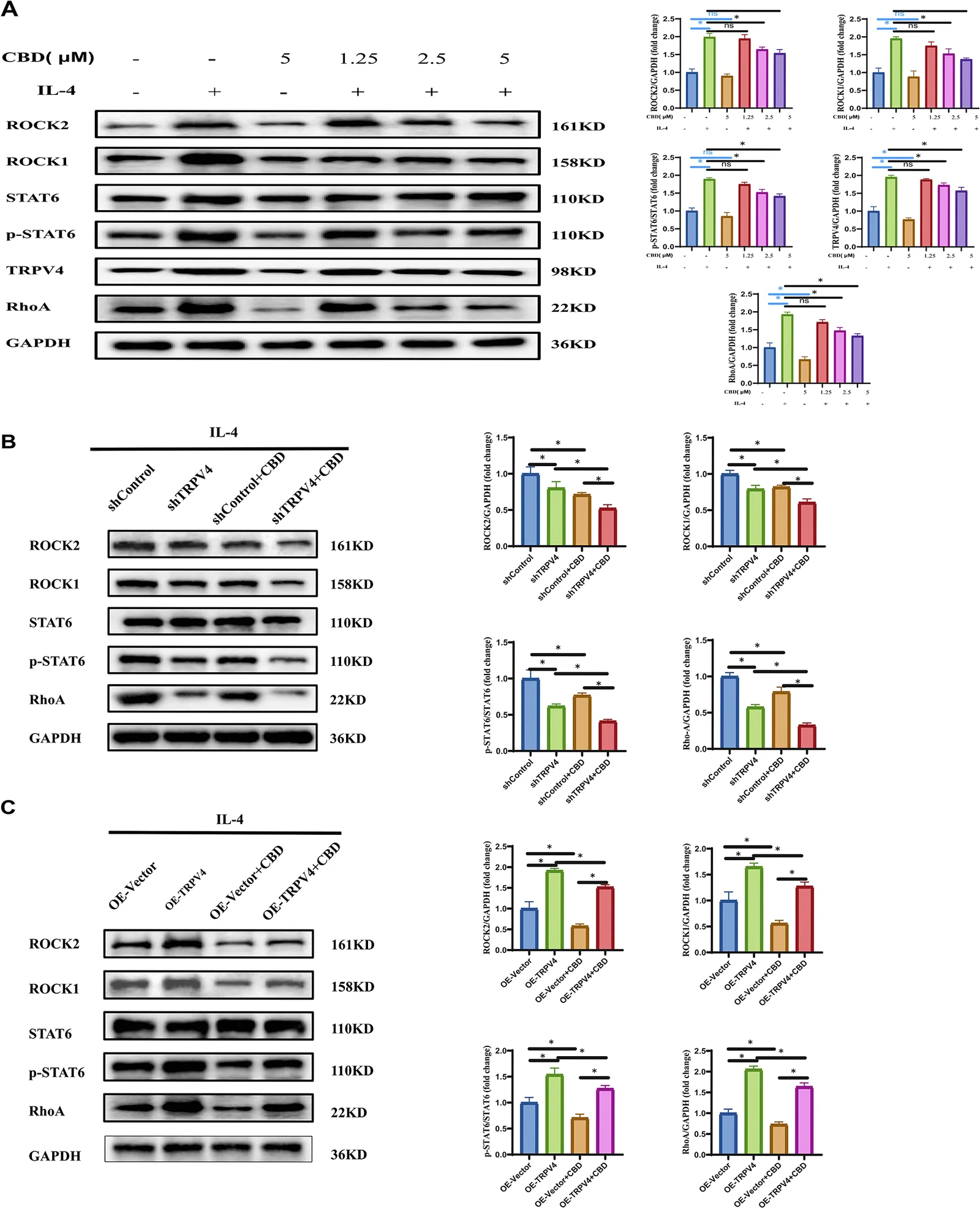
CBD suppresses the TRPV4/RhoA/ROCK/STAT6 signaling axis in IL-4-stimulated THP-1-derived macrophages. **(A)** Immunoblotting quantification of TRPV4, RhoA, ROCK1, ROCK2, STAT6, and p-STAT6 in IL-4-treated macrophages exposed to serially increasing CBD concentrations. **(B, C)** Immunoblotting detection of core signaling proteins in TRPV4-knockdown **(B)** or TRPV4-overexpressing **(C)** macrophages under IL-4 stimulation with or without CBD treatment. Data are presented as mean ± SEM (n = 3 per group). ^*^P < 0.05 indicates statistically significant differences; ns, non-significant.

In gain-of-function experiments, TRPV4 overexpression (OE-TRPV4) markedly increased the expression of RhoA, ROCK1, ROCK2, and p-STAT6 compared with the OE-Vector control (*P* < 0.05). CBD treatment effectively reversed these activations (**Figure 11B**). Conversely, in loss-of-function experiments, TRPV4 knockdown (shTRPV4) significantly reduced the levels of RhoA, ROCK1, ROCK2, and p-STAT6 (*P* < 0.05). CBD treatment mimicked the inhibitory effect of TRPV4 knockdown, and the combination of shTRPV4 and CBD further downregulated the pathway (**Figure 11C**).

Taken together, these results demonstrate that TRPV4 acts upstream of the RhoA/ROCK and STAT6, and that CBD inhibits M2-like polarization by targeting this signaling cascade.

### 3.13 Regulation of M2-like polarization by TRPV4-specific agonist and antagonist

The TRPV4 agonist GSK1016790A (0.5 μM) and antagonist RN-1734 (5 μM) were selected based on CCK-8 assays showing no cytotoxicity (**Figures 12A, B**). Consistently, TRPV4 and M2-associated marker mRNAs (MRC1, CD163) were upregulated in M2 macrophages, further enhanced by GSK101, and reduced by RN-1734 or CBD; similar trends were observed for M2-derived cytokines (IL-10, TGF-β) (**Supplementary Figure 1B**). Compared with the M2 group, GSK1016790A significantly increased the abundances of M2-associated markers (CD206, Arg-1, TGF-β, IL-10) and activated the TRPV4/RhoA/ROCK/STAT6 signaling axis, mimicking the effect of TRPV4 overexpression. In contrast, RN-1734 decreased these parameters, consistent with the effect of TRPV4 knockdown. Notably, CBD showed an inhibitory effect comparable to RN-1734 (*P* > 0.05) (**Figure 12C**). Immunofluorescence staining further confirmed that IL-4 stimulation markedly increased CD206 fluorescence intensity compared with the M0 group, whereas treatment with either RN-1734 or CBD significantly reduced CD206 fluorescence (*P* < 0.05), indicating that both agents effectively suppress M2-like macrophage polarization (**Figure 12D**).

**Figure 12:**
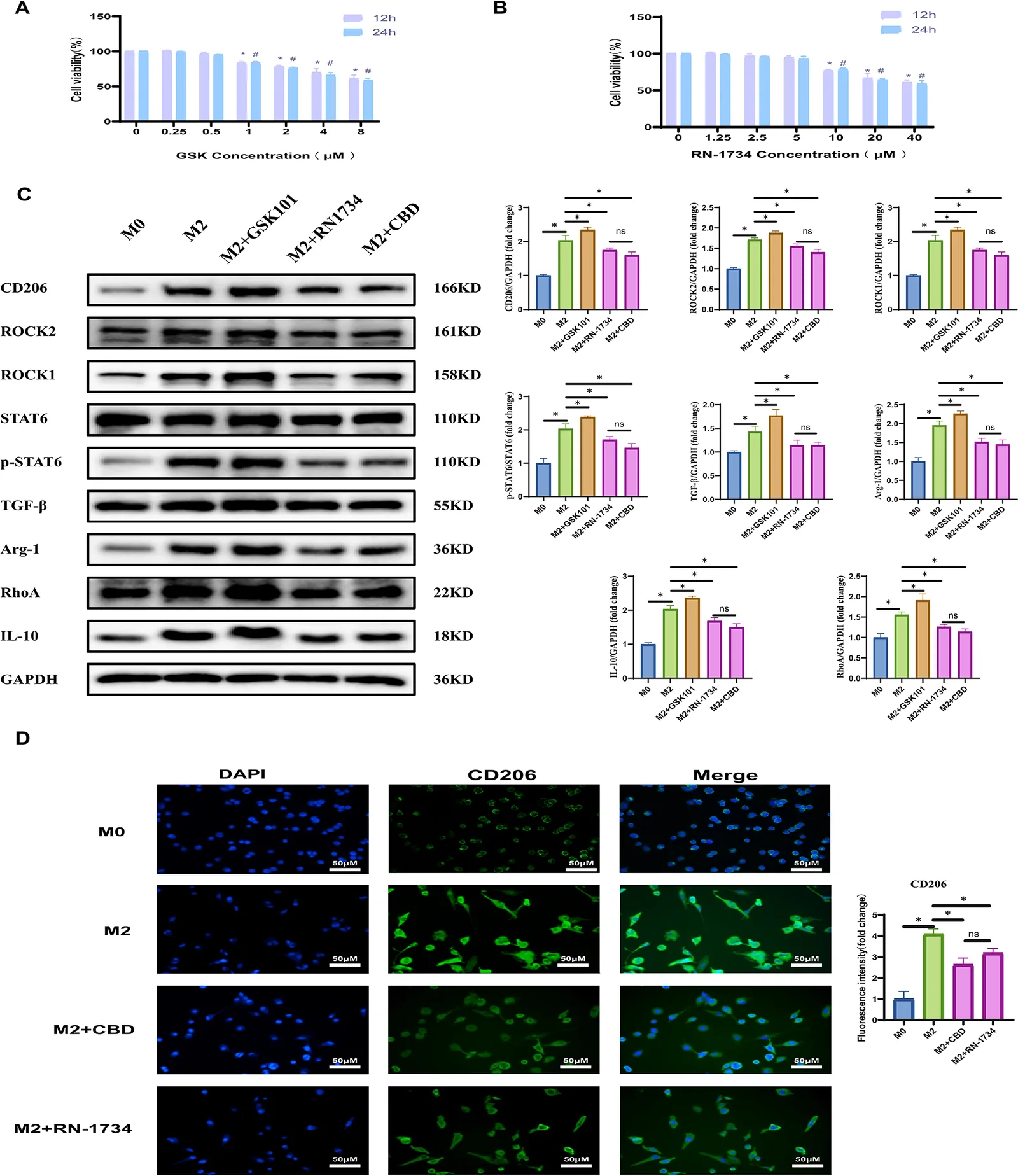
CBD blunts M2-like polarization by targeting TRPV4- RhoA/ROCK/STAT6 signaling in THP-1-derived macrophages. **(A, B)** CCK-8 assay assessing cell viability of THP-1-derived macrophages incubated with TRPV4 agonist GSK101 or antagonist RN-1734 at gradient concentrations for 12 h and 24 h. **(C)** Immunoblotting analysis of TRPV4/RhoA/ROCK/STAT6 cascade proteins and M2-associated markers in IL-4-stimulated macrophages treated with CBD, GSK101, or RN-1734. **(D)** Immunofluorescent staining visualization of CD206 expression in macrophages under different interventions (scale bar = 50 μm). Data are presented as mean ± SEM (n = 3 per group). ^*^P < 0.05 indicates statistically significant differences; ns, non-significant.

### 3.14 CBD regulates M2-like macrophage paracrine function to suppress HSCs activation

Conditioned medium (CM) derived from M2-like macrophages subjected to different treatments was collected and applied to LX-2 cells (**Figure 13A**). Compared with M0-CM, M2-CM significantly increased LX-2 cell migration, α-SMA fluorescence intensity, and the protein levels of Col-I, α-SMA, and TGF-β (all *P* < 0.05). In contrast, both M2+CBD-CM and M2+RN-1734-CM reversed these profibrotic effects (**Figures 13B–E**). Colony formation assays showed that shTRPV4-CM suppressed LX-2 colony formation, whereas OE-TRPV4-CM promoted it (*P* < 0.05, **Figure 13F**). Western blot analysis demonstrated that both M2-shTRPV4-CM and M2-shControl+CBD-CM significantly reduced the expression of fibrotic markers, with the combination (M2-shTRPV4+CBD-CM) exerting a more pronounced inhibitory effect (**Figure 13G**). Conversely, M2-OE-TRPV4-CM upregulated these markers, and this upregulation was reversed by CBD (**Figure 13H**). Collectively, these findings demonstrate that TRPV4 is a critical mediator of M2-like macrophage paracrine-induced HSC activation, and that CBD inhibits this process via TRPV4-associated signaling.

**Figure 13:**
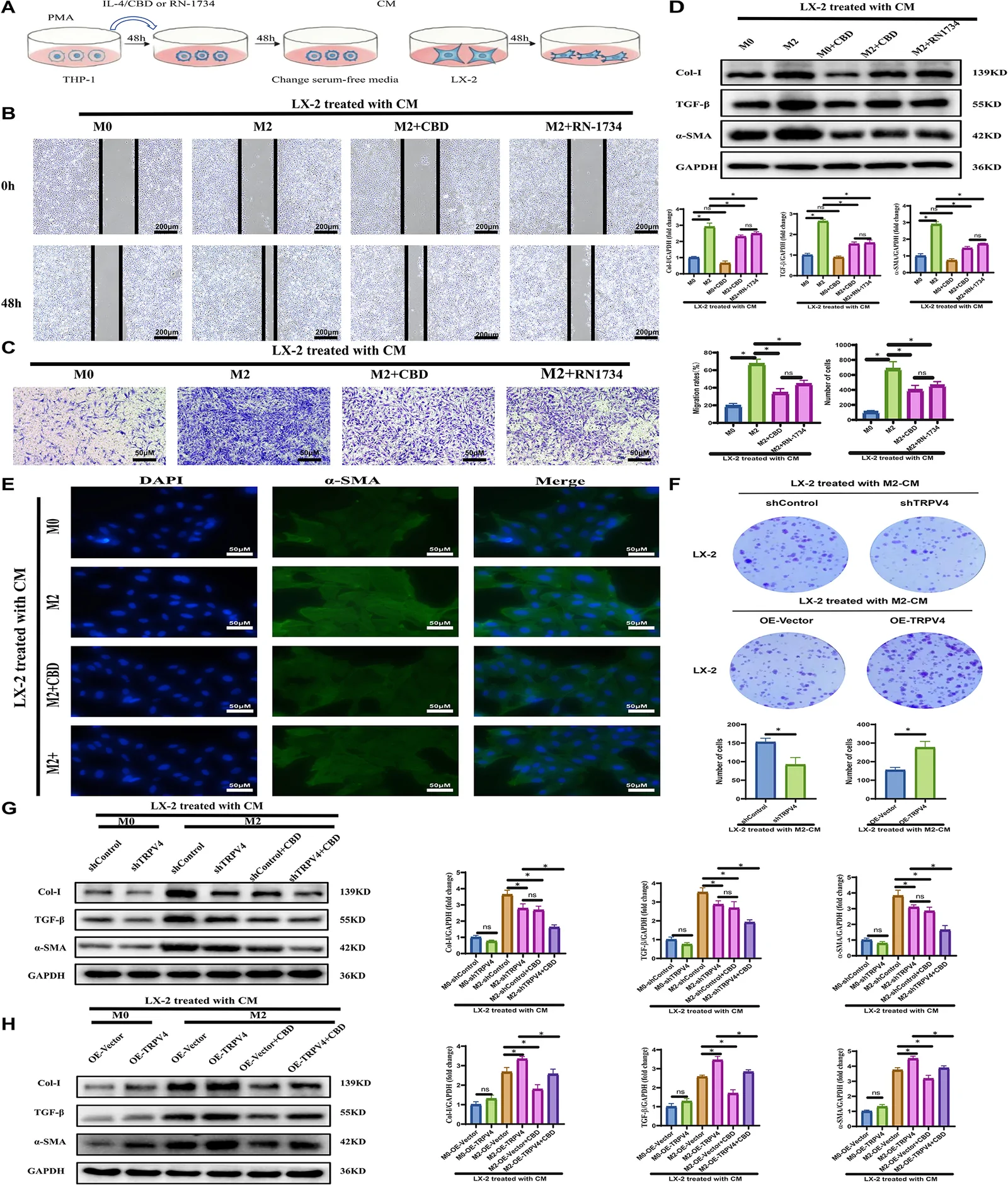
CBD inhibits LX-2 hepatic stellate cell activation by regulating TRPV4-mediated M2-like macrophage paracrine signaling. **(A)** Schematic diagram of the indirect co-culture system, in which conditioned medium (CM) harvested from THP-1-derived macrophages was applied to LX-2 hepatic stellate cells. **(B, C)** Wound-healing **(B)** and Transwell migration **(C)** assays demonstrated that CM from IL-4-stimulated macrophages treated with CBD or RN-1734 suppressed LX-2 cell migratory capacity (scale bar = 200 μm). **(D)** Immunoblotting quantification of fibrotic markers (Col-I, TGF-β, α-SMA) in LX-2 cells exposed to different macrophage-derived CM. **(E)** Immunofluorescent staining detection of α-SMA expression in LX-2 cells (scale bar = 50 μm); **(F–H)** Colony formation assay **(F)** and immunoblotting analysis of fibrotic proteins **(G, H)** in LX-2 cells cultured with CM from TRPV4-knockdown or TRPV4-overexpressing macrophages, with or without CBD treatment. Data are presented as mean ± SEM (n = 3 per group). ^*^P < 0.05 indicates statistically significant differences; ns, non-significant.

### 3.15 Pharmacological TRPV4 inhibition potentiates the hepatoprotective effects of cannabidiol against BDL-induced fibrogenic progression

To verify the relationship between CBD’s *in-vivo* antifibrotic effects and the TRPV4 pathway, BDL-induced mice were subjected to CBD monotherapy or co-treatment with RN-1734, a specific TRPV4 inhibitor. Relative to the BDL-injured cohort, monotherapies using either CBD or RN-1734 ameliorated macroscopic hepatic morphology, alleviated collagen accumulation, and diminished serum AST, ALT, and ALP concentrations (*P* < 0.05). The combination treatment further enhanced these beneficial effects (*P* < 0.05) (**Figures 14A–C**). Immunohistochemistry showed decreased expression of α-SMA, Col-I, F4/80, and CD163 with single-agent treatment, and the combination yielded even greater reductions (*P* < 0.05) (**Figure 14D**). Immunofluorescence staining confirmed that the combination treatment markedly diminished TRPV4/CD206 colocalization in liver tissues (**Figure 14E**). These results demonstrate that systemic pharmacological inhibition of TRPV4 synergistically potentiates the hepatoprotective effect of CBD in BDL-induced liver fibrosis, suggesting that the TRPV4 pathway represents a relevant target associated with CBD’s antifibrotic action.

**Figure 14:**
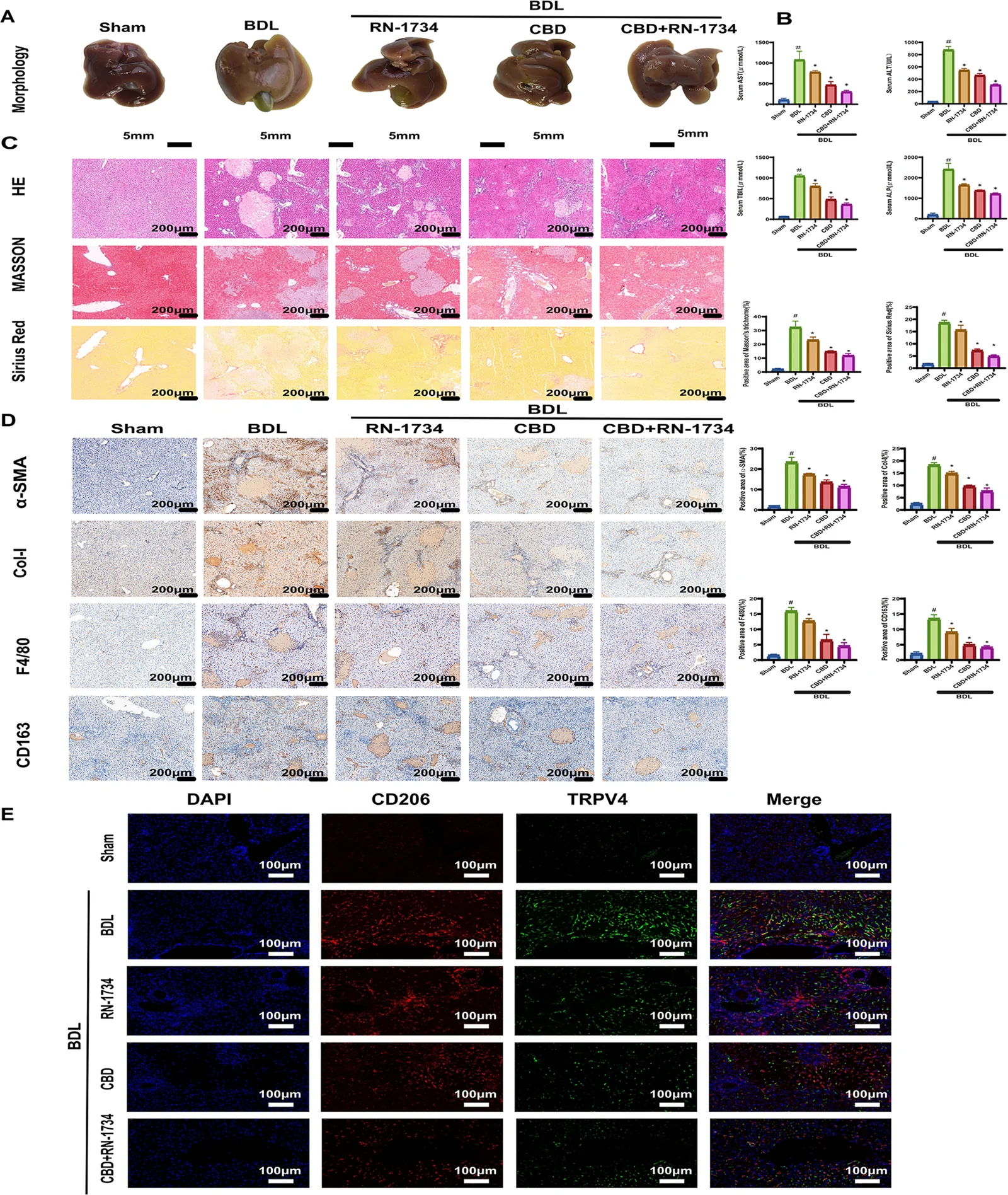
CBD alleviates BDL-induced hepatic fibrogenesis via repressing TRPV4-driven M2-like macrophage polarization *in vivo*. **(A)** Representative macroscopic hepatic morphology of mice from distinct experimental cohorts (scale bar = 5 mm). Groups: Sham (sham-operated), BDL (bile duct-ligated), BDL/RN-1734 (30 mg/kg RN-1734), BDL/CBD (8 mg/kg CBD), and BDL/CBD+RN-1734 (8 mg/kg CBD + 30 mg/kg RN-1734). **(B)** Serum levels of hepatic function biomarkers (ALP, TBIL, ALT, AST) in each group. **(C)** H&E, Masson’s trichrome, and Sirius Red staining evaluated hepatic pathological injury and collagen accumulation (scale bar = 200 μm). **(D)** IHC detection of fibrotic markers (α-SMA, Col-I) and macrophage markers (F4/80, CD163) in hepatic tissues (scale bar = 200 μm). **(E)** Immunofluorescent staining analysis of the co-localization pattern of M2-associated macrophage marker CD206 and TRPV4 in mouse liver sections (DAPI for nuclear counterstaining). Data are presented as mean ± SEM (n = 6 per group). *^#^P* < 0.05 versus the Sham group; ^*^P < 0.05 versus the BDL group.

## 4 Discussion

Liver fibrosis is increasingly recognized as a dynamic process influenced by the immune microenvironment, rather than solely as a consequence of chronic hepatic injury (23). Although hepatic stellate cells (HSCs) are central to extracellular matrix deposition, accumulating evidence suggests that macrophage-derived signals are critical in determining whether injured liver tissue progresses toward fibrogenesis or resolution (24). In this context, the present study identifies CBD as a potent modulator of the fibrotic immune niche (25). The data demonstrate that CBD attenuates BDL-induced liver injury and collagen accumulation, while mechanistically suppressing M2-like macrophage polarization via the TRPV4/RhoA/ROCK/STAT6 signaling axis. These observations offer novel perspectives on the antifibrotic pharmacological mechanisms of CBD and underscore TRPV4 as a previously undervalued immunomodulatory target in hepatic fibrosis.

Accumulating preclinical evidence highlights CBD-mediated anti-inflammatory, antioxidative, and metabolic-regulatory capacities in animal models of hepatic pathologies (26). Research has highlighted its ability to ameliorate steatosis, oxidative stress, and cytokine production (27). Nevertheless, scarce mechanistic insights have been gained into how cannabidiol modulates discrete immune subsets in fibrotic hepatic microenvironments. The present findings extend current understanding by demonstrating that CBD significantly alters the composition and activation status of hepatic macrophages. Although BDL injury increased both M1- and M2-like macrophages, CBD more selectively suppressed the accumulation of M2-polarized cells and their associated markers, including Arg-1, CD206, Retnla, Mrc1, and Chil3. This selectivity is mechanistically relevant because, in chronic liver disease, M2-like macrophages, despite their reparative features, can adopt a pro-fibrotic phenotype that secretes TGF-β and supports sustained HSC activation. This observation is consistent with emerging concepts in fibrosis research, which suggest that macrophage plasticity can become maladaptive when injury persists. Notably, these findings differ from some reports emphasizing CBD’s broad anti-inflammatory effects and instead suggest a more specialized immunomodulatory action directed at the M2-like lineage. Such differences may be attributable to disease model characteristics, as cholestatic fibrosis presents distinct macrophage transcriptional programs compared to metabolic or toxin-induced fibrosis. Nevertheless, the preferential inhibition of M2-like macrophages indicates that CBD disrupts the specific immune circuitry driving HSC activation.

A significant contribution of this study is the identification of TRPV4 as a functionally relevant molecular target of CBD in macrophages. Although TRPV4 has been implicated in several fibrotic disorders, its immune regulatory roles remain less well understood (28). The data indicate that TRPV4 is primarily expressed in Kupffer cells and lipid-associated macrophages, with this pattern becoming more pronounced in fibrosis. This cell-type specificity is notable, as most previous research has focused on TRPV4 in structural cells such as fibroblasts or epithelial cells. Single-cell analysis further revealed that TRPV4 expression correlated positively with M2-associated genes, but not with M1 markers, suggesting a potential role for TRPV4 in maintaining the pro-remodeling macrophage phenotype. Computational docking, molecular dynamics analysis, and CETSA collectively supported direct CBD–TRPV4 binding. This structural evidence, together with functional pharmacology, suggests that CBD may act as a TRPV4 inhibitor in macrophages. Existing literature indicates that TRPV4 regulates macrophage responses in a context-dependent manner, sometimes enhancing oxidative burst or bacterial clearance (29). The present findings expand this understanding by demonstrating that, under cholestatic injury, TRPV4 activation supports M2-like polarization. This divergence likely reflects the distinct metabolic and mechanical microenvironments across disease models, emphasizing the importance of tissue context when interpreting the immunological functions of TRPV4.

A key mechanistic insight from this study is that TRPV4 exerts its pro-fibrotic function through direct interaction with RhoA, thereby activating the RhoA/ROCK pathway and ultimately enhancing STAT6 phosphorylation, the master transcription factor for IL-4-induced M2-like polarization. The identification of a physical TRPV4–RhoA interaction in macrophages addresses an important gap in current knowledge, as most evidence linking TRPV4 to cytoskeletal regulation has been derived from non-immune cell types. Gain- and loss-of-function experiments convincingly demonstrate the upstream role of TRPV4 in RhoA/ROCK/STAT6 activation. CBD mimicked the effects of TRPV4 knockdown and effectively reversed the effects of TRPV4 overexpression, while TRPV4 agonism promoted M2-like polarization and TRPV4 antagonism replicated the suppressive effects of CBD. This hierarchical relationship suggests that CBD acts within a defined mechanotransduction signaling axis, rather than broadly inhibiting cellular activation. In addition to TRPV4, CBD is a polypharmacological compound that interacts with multiple molecular targets, including CB1/CB2 receptors, GPR55, PPARγ, 5-HT_1_A receptors, and adenosine signaling pathways (30). These targets may also contribute to the anti-fibrotic and anti-inflammatory effects observed in this study. In particular, PPARγ activation and GPR55 antagonism have been reported to modulate RhoA/ROCK signaling (31).

In addition to its direct effect on macrophage polarization, CBD significantly disrupted M2-like macrophage-derived paracrine signals that activate LX-2 cells (32). Conditioned medium from CBD- or TRPV4-inhibited macrophages reduced HSC migration, proliferation, and expression of fibrotic markers, whereas TRPV4 activation enhanced these features. These results underscore the central role of macrophage–HSC communication in sustaining fibrosis and demonstrate that the anti-fibrotic activity of CBD arises not only from suppressing fibrogenic mediators but also from reprogramming upstream immune signals.

Analysis of human fibrotic liver tissues revealed upregulation of TRPV4/RhoA/ROCK signaling and increased M2-like macrophage infiltration, underscoring the clinical relevance of this pathway. Although these data are correlative, they support the notion that TRPV4-associated macrophage polarization may represent a common mechanism across different etiologies of liver fibrosis. The enhanced therapeutic efficacy observed when CBD was combined with a TRPV4 antagonist *in vivo* further strengthens this translational potential, suggesting that TRPV4-directed strategies, whether through phytochemicals or synthetic inhibitors, could represent viable therapeutic avenues. Notably, as RN-1734 was systemically administered, our *in vivo* data do not distinguish TRPV4 activity in macrophages from that in other hepatic cell types, and our conclusions therefore pertain to systemic TRPV4 inhibition.

## 5 Conclusion

Collectively, these findings demonstrate that CBD attenuates liver fibrosis by modulating macrophage mechanotransduction. CBD may engage TRPV4, inhibit the TRPV4/RhoA/ROCK/STAT6 pathway, suppress M2-like macrophage polarization, and disrupt macrophage HSC crosstalk. The present investigation expands mechanistic insights into CBD as an immunoregulatory phytocompound and pinpoints TRPV4-associated macrophage reprogramming as a viable pharmacological target for hepatic fibrogenesis.

## Data Availability

The datasets presented in this study can be found in the online repository: NCBI: Accession number: GSE136103.

## References

[1] Taru V Szabo G Mehal W Reiberger T . Inflammasomes in chronic liver disease: hepatic injury, fibrosis progression and systemic inflammation. J Hepatol. (2024) 81:895–910. doi: 10.1016/j.jhep.2024.06.016 38908436 PMC11881887

[2] Khanam A Saleeb PG Kottilil S . Pathophysiology and treatment options for hepatic fibrosis: can it be completely cured? Cells. (2021) 10:1097. doi: 10.3390/cells10051097 34064375 PMC8147843

[3] Bourebaba N Marycz K . Hepatic stellate cells role in the course of metabolic disorders development - a molecular overview. Pharmacol Res. (2021) 170:105739. doi: 10.1016/j.phrs.2021.105739 34171492

[4] Merens V Knetemann E Gürbüz E De Smet V Messaoudi N Reynaert H . Hepatic stellate cell single cell atlas reveals a highly similar activation process across liver disease aetiologies. JHEP Rep Innov Hepatol. (2025) 7:101223. doi: 10.1016/j.jhepr.2024.101223 39758511 PMC11699746

[5] Xie D Huang Y Yu Y Jin W Zhang X Yu F . Interactions between hepatic stellate cells and immune cells: implications for liver fibrosis. Biochim Biophys Acta Mol Basis Dis. (2026) 1872:168062. doi: 10.1016/j.bbadis.2025.168062 41056595

[6] Tang X Bai M Du X Wang H Liu M Fu X . Novel regulators of hepatic macrophages in liver fibrosis. Front Immunol. (2025) 16:1705503. doi: 10.3389/fimmu.2025.1705503 41479922 PMC12754181

[7] Zhang J Xie Z Zhu X Xu C Lin J Zhao M . New insights into therapeutic strategies for targeting hepatic macrophages to alleviate liver fibrosis. Int Immunopharmacol. (2025) 158:114864. doi: 10.1016/j.intimp.2025.114864 40378438

[8] Bai L Liu X Zheng Q Kong M Zhang X Hu R . M2-like macrophages in the fibrotic liver protect mice against lethal insults through conferring apoptosis resistance to hepatocytes. Sci Rep. (2017) 7:10518. doi: 10.1038/s41598-017-11303-z 28874845 PMC5585332

[9] Jiang Y Cai R Huang Y Zhu L Xiao L Wang C . Macrophages in organ fibrosis: from pathogenesis to therapeutic targets. Cell Death Discov. (2024) 10:487. doi: 10.1038/s41420-024-02247-1 39632841 PMC11618518

[10] Yang F Duan S Liu J An Z Liu W Wang X . Antitumor effects of cannabidiol (CBD) on osteosarcoma by targeting TNF-α/NF-κB/CCL5 signaling axis. Phytomedicine Int J Phytother Phytopharm. (2025) 145:157066. doi: 10.1016/j.phymed.2025.157066 40680332

[11] Chen S Kim J-K . The role of cannabidiol in liver disease: a systemic review. Int J Mol Sci. (2024) 25:2370. doi: 10.3390/ijms25042370 38397045 PMC10888697

[12] Krzyżewska A Baranowska-Kuczko M Kasacka I Kozłowska H . Cannabidiol alleviates right ventricular fibrosis by inhibiting the transforming growth factor β pathway in monocrotaline-induced pulmonary hypertension in rats. Biochim Biophys Acta Mol Basis Dis. (2023) 1869:166753. doi: 10.1016/j.bbadis.2023.166753 37187449

[13] Sun X Zhou L Wang Y Deng G Cao X Ke B . Single-cell analyses reveal cannabidiol rewires tumor microenvironment via inhibiting alternative activation of macrophage and synergizes with anti-PD-1 in colon cancer. J Pharm Anal. (2023) 13:726–44. doi: 10.1016/j.jpha.2023.04.013 37577382 PMC10422166

[14] Sánchez-Hernández R Benítez-Angeles M Hernández-Vega AM Rosenbaum T . Recent advances on the structure and the function relationships of the TRPV4 ion channel. Channels Austin Tex. (2024) 18:2313323. doi: 10.1080/19336950.2024.2313323 38354101 PMC10868539

[15] Adapala RK Katari V Teegala LR Thodeti S Paruchuri S Thodeti CK . TRPV4 mechanotransduction in fibrosis. Cells. (2021) 10:3053. doi: 10.3390/cells10113053 34831281 PMC8619244

[16] Grace MS Bonvini SJ Belvisi MG McIntyre P . Modulation of the TRPV4 ion channel as a therapeutic target for disease. Pharmacol Ther. (2017) 177:9–22. doi: 10.1016/j.pharmthera.2017.02.019 28202366

[17] Liu J Guo Y Zhang R Xu Y Luo C Wang R . Inhibition of TRPV4 remodels single cell polarity and suppresses the metastasis of hepatocellular carcinoma. Cell Death Dis. (2023) 14:379. doi: 10.1038/s41419-023-05903-z 37369706 PMC10300155

[18] Wang J Yuan Z Yu N Jiao Q Zhou H Liao W . Inactivation of RhoA for hypertension treatment through the TRPV4-RhoA-RhoGDI1 axis. Circulation. (2025) 152:519–36. doi: 10.1161/CIRCULATIONAHA.124.071884 40518994

[19] Luo C Huang C Zhu Y Zhou Y Qiao Y Shi C . Inhibition of Rho GEFs attenuates pulmonary fibrosis through suppressing myofibroblast activation and reprogramming profibrotic macrophages. Cell Death Dis. (2025) 16:278. doi: 10.1038/s41419-025-07573-5 40216763 PMC11992128

[20] Dutta SD An JM Mondal J Bose S Jana SK Seol Y . Ion channel/Stat6-driven nano-immune programming of tissue-resident macrophages by amide-functionalized nanocellulose. Bioact Mater. (2026) 62:883–98. doi: 10.1016/j.bioactmat.2026.03.038 42004621 PMC13091342

[21] Ma R Xie N Shu Y Wu Y He P Xiang Y . Cannabidiol alleviates carbon tetrachloride-induced liver fibrosis in mice by regulating NF-κB and PPAR-α pathways. Exp Biol Med Maywood NJ. (2024) 249:10141. doi: 10.3389/ebm.2024.10141 38711461 PMC11070938

[22] Wang J Guo T Peng Q-S Yue S-W Wang S-X . Berberine via suppression of transient receptor potential vanilloid 4 channel improves vascular stiffness in mice. J Cell Mol Med. (2015) 19:2607–16. doi: 10.1111/jcmm.12645 26177349 PMC4627566

[23] Widowati W Sabrina AHN Sutendi AF Zahiroh FH Nurjamil AM Wargasetia TL . Immune system and hepatic stellate cells’ crosstalk in liver fibrosis: pathways and therapeutic potential. J Immunol Res. (2026) 2026:2656395. doi: 10.1155/jimr/2656395 41487942 PMC12759114

[24] SchwabeRF BrennerDA . Hepatic stellate cells: balancing homeostasis, hepatoprotection and fibrogenesis in health and disease. Nat Rev Gastroenterol Hepatol. (2025) 22:481–99. doi: 10.1038/s41575-025-01068-640404839

[25] Luo D Zhang J Yin H Li S Xu S Li S . Cannabidiol alleviates perfluorooctane sulfonate-induced macrophage extracellular trap mediate inflammation and fibrosis in mice liver. Ecotoxicol Environ Saf. (2023) 263:115374. doi: 10.1016/j.ecoenv.2023.115374 37591127

[26] Peng J Fan M An C Ni F Huang W Luo J . A narrative review of molecular mechanism and therapeutic effect of cannabidiol (CBD). Basic Clin Pharmacol Toxicol. (2022) 130:439–56. doi: 10.1111/bcpt.13710 35083862

[27] Burstein S . Cannabidiol (CBD) and its analogs: a review of their effects on inflammation. Bioorg Med Chem. (2015) 23:1377–85. doi: 10.1016/j.bmc.2015.01.059 25703248

[28] Michalick L Kuebler WM . TRPV4-a missing link between mechanosensation and immunity. Front Immunol. (2020) 11:413. doi: 10.3389/fimmu.2020.00413 32210976 PMC7076180

[29] Nguyen T-N Siddiqui G Veldhuis NA Poole DP . Diverse roles of TRPV4 in macrophages: a need for unbiased profiling. Front Immunol. (2022) 12. doi: 10.3389/fimmu.2021.828115 35126384 PMC8811046

[30] Jeon K-H Kim S Park S Pak ES Kim S Lee HJ . Cannabigerol attenuates liver fibrosis via AMPK activation: regulation of lipid metabolism, inflammation, and hepatic stellate cell activation. BioMed Pharmacother Biomedecine Pharmacother. (2025) 191:118463. doi: 10.1016/j.biopha.2025.118463 40818359

[31] Nakajima K Oka S Tanikawa T Nemoto-Sasaki Y Matsumoto N Ishiguro H . Lysophosphatidylinositol induced morphological changes and stress fiber formation through the GPR55-RhoA-ROCK pathway. Int J Mol Sci. (2022) 23:10932. doi: 10.3390/ijms231810932 36142844 PMC9504244

[32] Wang Z Du K Jin N Tang B Zhang W . Macrophage in liver fibrosis: identities and mechanisms. Int Immunopharmacol. (2023) 120:110357. doi: 10.1016/j.intimp.2023.110357 37224653

