# Supplementary material for: Cannabidiol suppresses M2-like macrophage polarization via TRPV4/RhoA/ROCK signaling to alleviate hepatic fibrosis
Source: Front Immunol. 2026 Oct 20;17:1951332. doi: 10.3389/fimmu.2026.1951332 (PMC13727404; doi:10.3389/fimmu.2026.1951332)
Supplement: Supplementary file 1 [file SupplementaryFile1.docx]

# 1  Supplementary Data

**Supplementary Methods**

**Immunofluorescence staining**

Paraffin-embedded liver tissue sections or cultured cells were subjected to immunofluorescence staining following standardized procedures. After deparaffinization and rehydration of tissue sections or fixation of cultured cells with 4% paraformaldehyde for 15 minutes, all samples were permeabilized with 0.3% Triton X-100 for 23 minutes, then blocked with 3% BSA at room temperature for 90 minutes. Subsequently, the samples were incubated overnight at 4°C with specific primary antibodies, followed by incubation with fluorescence-conjugated secondary antibodies for 1–2 hours at room temperature in the dark. Nuclei were counterstained with DAPI, and images were captured using a Nikon inverted fluorescence microscope to observe the localization and expression of target proteins.

**PCR（RNA Extraction, Reverse Transcription, and qRT-PCR）**

Total RNA was extracted from approximately 35 mg of liver tissue or cultured cells using RNAiso reagent. The extraction process included grinding the samples in liquid nitrogen, followed by lysis, centrifugation, isopropanol precipitation, ethanol washing, and dissolution in RNase-free water to obtain high-purity total RNA. Reverse transcription was performed using the HP All-in-one qRT Master Mix II kit (Yunyun Heng, China), with a 20 μL reaction system containing 1 μg of total RNA. The reaction program was set as follows: 50°C for 5 minutes (2 cycles) and 80°C for 5 seconds. For real-time quantitative PCR (qRT-PCR), a 20 μL reaction mixture was prepared, including 10 μL of 2× Universal SYBR qPCR Master Mix, 1 μL each of 10 μM forward and reverse primers, and 2 μL of cDNA template. Glyceraldehyde-3-phosphate dehydrogenase (GAPDH) was used as an internal reference. The primer sequences for all target genes and GAPDH are listed in Supplementary Table 1. The amplification protocol was: initial denaturation at 95°C for 30 seconds, followed by 40–45 cycles of denaturation at 95°C for 5 seconds and annealing/extension at 60°C for 10–30 seconds, and final melt curve analysis to verify the specificity of the amplification product. Relative gene expression levels were calculated using the 2^−ΔΔCt method.

**Cellular thermal shift assay (CETSA)**

CETSA was performed following the protocol of Martinez Molina et al. (2013). Briefly, cells were divided into two groups: the CBD+ group was treated with CBD, and the CBD– group received PBS as a vehicle control. Cells were then harvested and adjusted to 1 × 10⁷ cells/mL. Cell suspensions were heated individually at the indicated temperatures for 3 min and immediately cooled on ice. Cells were lysed by freeze-thawing in liquid nitrogen, and the lysates were centrifuged to collect supernatants. The soluble supernatant was further centrifuged for 20 min and analyzed by Western blotting.

# 2 Supplementary Figures and Tables

## 2.1 Supplementary Figures

**Supplementary Figure 1.** RT-qPCR detection of macrophage polarization markers and cytokines. (A) The mRNA relative expression of Nos2, Tnf-α, Il-10, Tgf-β, Cd9, Trem2, and Ssp1 in liver tissue from Sham, BDL, CBD-L, CBD-H and Silymarin groups. # vs. Sham; * vs. BDL. (B) mRNA levels of TRPV4, M2 markers (MRC1, CD163), Il-10 and Tgf-β in THP-1-derived M0, M2 macrophages, and M2 macrophages intervened with GSK101, RN-1734 or CBD. **p* < 0.05; ns, not significant

**Supplementary Figure 2.** Comparison of TRPV4 expression in hepatic macrophage subpopulations from CON control and MOD fibrotic mouse livers based on single-cell RNA-seq data.(A) Split UMAP feature plots mapping TRPV4 expression in macrophages of CON healthy mice (left) and MOD fibrotic mice (right), with warmer colors indicating higher TRPV4 transcript levels.

(B) Heatmap displaying the average TRPV4 expression value of each macrophage subtype in CON and MOD groups, color-coded by normalized expression intensity.

**Supplementary Figure 3.** Bulk RNA-seq analysis of human normal and fibrotic liver tissues. RNA-seq of 3 normal and 3 fibrotic human livers characterizes fibrosis-related transcriptional changes. (A) Expression heatmap of all differentially expressed genes (DEGs) between Normal and Fibrosis groups. (B) GO biological process enrichment dot plot of all DEGs. (C) Bar charts summarizing the top 10 enriched GO terms across three GO subclasses. (D) KEGG pathway enrichment bubble plot based on all DEGs. (E) Heatmap of TRPV4-related significant DEGs, classified by gene functional features. (F) Bar statistics of upregulated and downregulated DEGs in calcium signaling and TRPV4 downstream gene categories. (G) Correlation scatter plot showing Pearson correlations between TRPV4 expression and its associated key DEGs.

**Supplementary Figure 4.** Fluo-4 AM staining showing Ca²⁺ influx in M2 cells. M2 macrophages were treated with vehicle (M2), GSK101 (M2+GSK101), or GSK101 + CBD (M2+GSK101+CBD). Ca²⁺ influx was detected by Fluo-4 AM (green), nuclei by DAPI (blue). Merged images are shown; scale bar = 100 μm.

## 2.2 Supplementary Table

**Tab. 1 Primer sequences**

| Gene name | Direction | Sequences（5’ to 3’） |
| --- | --- | --- |
| *Arg1* | F | TCTGCCAAAGACATCGTGTACAT |
|  | R | CGACATCAAAGCTCAGGTGAATC |
| *Retnla* | F | CCTCCACTGTAACGAAGACTCTC |
|  | R | CTCCCAAGATCCACAGGCAAAG |
| *Mrc1* | F | GTGGACGCTCTAAGTGCCAT |
|  | R | GAATCTGACACCCAGCGGAA |
| *Il-10* | F | ATTTGAATTCCCTGGGTGAGAAG |
|  | R | CACAGGGGAGAAATCGATGACA |
| *Nos2* | F | ATCTTTGCCACCAAGATGGCCTGG |
|  | R | TTCCTGTGCTGTGCTACAGTTCCG |
| *Tgf-β* | F | CTCCCGTGGCTTCTAGTGC |
|  | R | GCCTTAGTTTGGACAGGATCTG |
| *Tnf-α* | F | GTCCCCAAAGGGATGAGAAGT |
|  | R | TTTGCTACGACGTGGGCTAC |
| *Trpv4* | F | TCACCTTCGTGCTCCTGTTG |
|  | R | AGATGTGCTTGCTCTCCTTG |
| *Chi3* | F | ATGAAGCATTGAATGGTCTGAAAG |
|  | R | TGAATATCTGACGGTTCTGAGGAG |
| *Gapdh* | F | TGTGTCCGTCGTGGATCTGA |
|  | R | TTGCTGTTGAAGTCGCAGGAG |
| *TRPV4* | F | GATGGGCGACCAAATCTGC |
|  | R | GAGGACTCATATAGGGTGGACTC |
| *CD163* | F | TTTGTCGCTTGAAGTCCCTTCAC |
|  | R | TCCCGCCTACTCTTGTTTTCAC |
| *MRC1* | F | GGGTTGCTATCACTCTCTATGC |
|  | R | TTTCTCGCTCTGTGCCGTAGTT |
| *TGF-β* | F | CTAATGGTGGAAACCCACAACG |
|  | R | TATCGC CAGGAATTGTTGCTG |
| *IL-10* | F | GACTTTTAAGGGTTACCTGGGTTG |
|  | R | TCACATGCGCCTTGATGTCTG |
| *GAPDH* | F | GGCGGGAAAGAAGATCCAC |
|  | R | GCAGACTTGACCAACTGTCATT |
| *Trem2* | F | CTGGAACCGTCACCATCACTC |
|  | R | CGAAACTCGATGACTCCTCGG |
| *Cd9* | F | TGGTCGCTGGTGGTGCTGTA |
|  | R | CAGTCGGAGCAGGTGATGGT |
| *Spp1* | F | GTCTGGAGAACATGGGTGCT |
|  | R | GGGTGCAGGCTGTAAAGCTA |

**Tab. 2 Baseline clinical and biochemical characteristics of study participants**

| **Variable** | **Total (n = 12)** | **Fibrosis (n = 6)** | **Normal (n = 6)** | **P-value** | **Statistic** |
| --- | --- | --- | --- | --- | --- |
| Age (years), median (IQR) | 55.00 (49.75–61.75) | 55.50 (46.25–62.25) | 54.50 (46.75–61.75) | 0.769 | Z= -0.274 |
| Sex (Male/Female), n (%) | 6 (50.0) / 6 (50.0) | 3 (50.0) / 3 (50.0) | 3 (50.0) / 3 (50.0) | 1.000 | χ²= 0.000 |
| AST (U/L), median (IQR) | 257.40 (128.23–524.17) | 297.70 (143.55–679.80) | 121.70 (24.20–162.70) | 0.010* | Z= -2.581 |
| ALT (U/L), median (IQR) | 192.50 (88.02–412.22) | 265.00 (100.35–558.30) | 129.60 (14.90–152.60) | 0.043* | Z= -2.023 |
| TBIL (μmol/L), median (IQR) | 24.96 (16.88–33.05) | 28.85 (18.25–41.52) | 13.70 (8.20–17.60) | 0.002* | Z= -3.088 |
| DBIL (μmol/L), median (IQR) | 9.95 (5.57–17.09) | 11.90 (8.65–25.20) | 4.70 (4.20–5.60) | < 0.001* | Z= -3.954 |
| PLT (×10⁹/L), median (IQR) | 131.50 (83.50–182.25) | 107.00 (72.50–150.50) | 188.00 (136.00–261.00) | 0.002* | Z= -3.051 |
| FIB (g/L), median (IQR) | 2.61 (1.90–3.23) | 2.44 (1.73–3.12) | 3.19 (2.45–3.38) | 0.060 | Z= -1.882 |
| Fibrosis stage (IV), n (%) | 6 (50.0) | 6 (100.0) | 0 (0.0) | < 0.001* | χ² = 12.000 |
| HBV infection, n (%) | 5 (41.7) | 5 (83.3) | 0 (0.0) | < 0.001* | χ² = 7.500 |
| Alcohol-related liver disease, n (%) | 1 (8.3) | 1 (16.7) | 0 (0.0) | 0.999 | χ² = 1.091 |
